# Molecular Approaches in Fetal Malformations, Dynamic Anomalies and Soft Markers: Diagnostic Rates and Challenges—Systematic Review of the Literature and Meta-Analysis

**DOI:** 10.3390/diagnostics12030575

**Published:** 2022-02-23

**Authors:** Gioia Mastromoro, Daniele Guadagnolo, Nader Khaleghi Hashemian, Enrica Marchionni, Alice Traversa, Antonio Pizzuti

**Affiliations:** Department of Experimental Medicine, Policlinico Umberto I Hospital, Sapienza University of Rome, 00161 Rome, Italy; daniele.guadagnolo@uniroma1.it (D.G.); nader.khaleghihashemian@uniroma1.it (N.K.H.); enrica.marchionni@uniroma1.it (E.M.); alice.traversa@uniroma1.it (A.T.); antonio.pizzuti@uniroma1.it (A.P.)

**Keywords:** molecular approaches, chromosomal microarray, exome sequencing, genome sequencing, prenatal diagnosis, genetic counseling, fetal malformations, structural anomalies, soft markers

## Abstract

Fetal malformations occur in 2–3% of pregnancies. They require invasive procedures for cytogenetics and molecular testing. “Structural anomalies” include non-transient anatomic alterations. “Soft markers” are often transient minor ultrasound findings. Anomalies not fitting these definitions are categorized as “dynamic”. This meta-analysis aims to evaluate the diagnostic yield and the rates of variants of uncertain significance (VUSs) in fetuses undergoing molecular testing (chromosomal microarray (CMA), exome sequencing (ES), genome sequencing (WGS)) due to ultrasound findings. The CMA diagnostic yield was 2.15% in single soft markers (vs. 0.79% baseline risk), 3.44% in multiple soft markers, 3.66% in single structural anomalies and 8.57% in multiple structural anomalies. Rates for specific subcategories vary significantly. ES showed a diagnostic rate of 19.47%, reaching 27.47% in multiple structural anomalies. WGS data did not allow meta-analysis. In fetal structural anomalies, CMA is a first-tier test, but should be integrated with karyotype and parental segregations. In this class of fetuses, ES presents a very high incremental yield, with a significant VUSs burden, so we encourage its use in selected cases. Soft markers present heterogeneous CMA results from each other, some of them with risks comparable to structural anomalies, and would benefit from molecular analysis. The diagnostic rate of multiple soft markers poses a solid indication to CMA.

## 1. Introduction

Fetal malformations are diagnosed in 2–3% of pregnancies [1,2,3,4,5], representing a significant public health concern, requiring further morphological examinations, genetic counseling, invasive procedures for cytogenetics and molecular testing, and appropriate obstetric/perinatal management [6]. Some ultrasonographic (US) investigations are usually planned. The first-trimester US scan, commonly performed at 11–13 weeks of gestational age (wga), assesses nuchal translucency (NT) and nasal bone presence, and excludes tricuspid regurgitation and ductus venosus reversal flow. The mid-trimester US scan (18–22 wga) consists of a systematic sequential study of fetal organs. US scan efficiency for the detection of anomalies spans from 15% to 85%, depending on the wga, the sonographer’s expertise, the woman’s body mass index and the involved fetal organ [7]. Different kinds of anomalies can be identified. Structural anomalies include non-transient anatomic alterations of organs or limbs development. Nonspecific and often transient minor US findings that cannot be considered as structural are called “soft markers” (increased NT, short femur, echogenic intracardiac focus, mild ventriculomegaly, enlarged cisterna magna, choroid plexus cysts, echogenic bowel, mild hydronephrosis, absent/hypoplastic nasal bone and single umbilical artery) [8,9,10,11]. Several papers consider increased NT as either a soft marker or structural anomaly or a separate entity. We categorized as “dynamic” those anomalies that can regress or worsen during pregnancies, not fitting either the soft marker or structural anomaly definition. These include quantitative changes of amniotic fluid (anhydramnios, oligohydramnios and polyhydramnios), fetal growth restriction (FGR), effusions (hydrops, pleural or pericardial effusions, ascites, hydroderma) and cystic hygroma. Isolated soft markers are noted in up to 10% of fetuses [12]. They are considered mainly risk factors for chromosomopathies, and invasive diagnostic testing is usually not recommended after the detection of an isolated soft marker, following low-risk results of screening evaluations for aneuploidies (combined first-trimester screen; non-invasive prenatal screening (NIPS)). Diagnostic approaches for US anomalies are improving, and genetic testing provides valuable information about the prognosis of the fetus, being crucial to proper genetic counseling, and can be necessary to discuss future reproductive options. Cytogenetics and molecular testing can be requested. Standard karyotyping consists in the visualization of the entire mitotic structures, identifying chromosomal anomalies of number and structure with resolution limits of 5–10 Mb.

Molecular investigations are performed on fetal DNA extracted from invasive samples (chorionic villus, amniotic fluid and fetal blood) [13]. Array-based molecular cytogenetic analyses (chromosomal microarray analysis (CMA)) permit the detection of even smaller chromosomal imbalances (copy number variations (CNVs)), reaching a resolution of 50 Kb. The main approaches used are array-comparative genomic hybridization (a-CGH) and single nucleotide polymorphism array (SNP-array). SNP-array analyzes the sample through hybridization with allele-specific oligonucleotide probes, additionally identifying runs of homozygosity (ROHs), which can be caused by consanguinity or uniparental disomy, and mosaicism at a lower percentage than a-CGH [14]. Next generation sequencing (NGS) approaches revolutionized clinical practice in medical genetics, identifying point mutations within a limited timeframe and becoming a staple of prenatal diagnosis. NGS applications are represented by targeted panels, exome sequencing (ES), and whole genome sequencing (WGS). Diagnostic ES is used in selected fetuses with US anomalies and negative cytogenetics testing. WGS is an emerging tool whose diagnostic potential is currently being explored, mainly in research settings.

This meta-analysis aims to evaluate the diagnostic yield, the rates of results of uncertain significance (variants of uncertain significance (VUSs)) and incidental findings (IFs), pathogenic variants in genes unrelated to the clinical indication, in cohorts of fetuses undergoing non-targeted molecular testing due to structural and dynamic anomalies and soft markers. This review highlights characteristics, limitations, implications and challenges of the main molecular techniques adopted in prenatal diagnosis assessments, pointing out the importance of contextualizing molecular findings within a multidisciplinary team of experts in fetal medicine.

## 2. Materials and Methods

**Figure 1:**
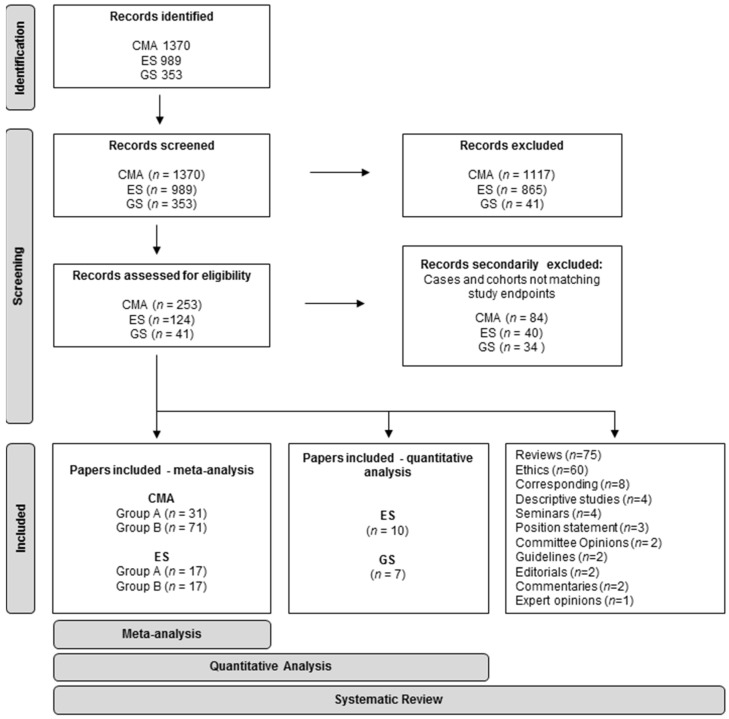
PRISMA flowchart of the systematic review and meta-analysis.

The research was conducted following PRISMA guidelines [15] (Figure 1). We searched the Pubmed database (https://pubmed.ncbi.nlm.nih.gov/, accessed on 12 November 2021) for “prenatal” AND/OR (specific string for the molecular technique), with a 10-year filter for publication date (1 January 2011–12 November 2021). All titles and abstracts were examined. Only papers with full text available in English language were retained. Case reports and papers not describing the prenatal diagnostic application of the specific molecular technique on invasive samples were excluded.

Prenatal CMA and ES cohorts deemed eligible for quantitative analysis were divided into two groups for each approach: Group A, including cohorts with pooled different indications, and Group B, including fetuses enrolled for a specific single-district anomaly. Cases with postnatal diagnosis, fetuses enrolled for known chromosomal/molecular variants and familiarity/recurrence for genetic disorders, cases enrolled after fetal demise and twin pregnancies were excluded. Papers in which these categories were included but could not be separated from eligible cases were secondarily excluded.

Cases from eligible papers were classified into categories and subcategories. For each cohort and category, the findings were classified as either single, multiple or pooled, regardless of association (identified as “pooled” or “any”). The categories were: no indication of invasive testing; advanced maternal age; soft marker(s); structural anomalies, single, multiple, or pooled, regardless of association; and dynamic anomalies. Subcategories identifying specific districts for structural anomalies were: central nervous system, musculoskeletal, genitourinary, gastrointestinal, cardiovascular, thorax/respiratory/chest, craniofacial, abdominal and body wall. Subcategories for dynamic anomalies were: amniotic fluid quantity anomalies (polyhydramnios and oligohydramnios), FGR and hydrops. Subcategories for soft markers were: echogenic intracardiac focus, mild ventriculomegaly, enlarged cisterna magna, choroid plexus cysts, echogenic bowel, mild hydronephrosis, absent/hypoplastic nasal bone, short femur and single umbilical artery.

Due to the inconsistency in the classification of NT as a soft marker, a structural anomaly, or an autonomous condition, we regarded it as independent. For each paper, we annotated study design and sample types, when available. Pathogenic and likely pathogenic variants relevant to the phenotype were considered as diagnostic and annotated as P/LP. We also annotated VUSs and IFs. IFs, when declared, were excluded from the count of diagnostic rate. We scored diagnostic and VUS yields for each specific indication and approach for each paper. The diagnostic yield was scored as (n. cases with P/LP variant identified)/(eligible cases), while VUS rate was (n. cases with VUS identified)/(eligible cases).

For prenatal WGS cohorts, because of the reduced number of studies and the different sensitivity of the approaches, we performed quantitative analysis without distinguishing between Group A and Group B.

The meta-analysis was carried out for the CMA and ES cohorts. It was performed by scoring diagnostic and VUS rates, pooling cases collected within the same category and undergoing the same testing from eligible papers. Standard deviations and 95% confidence intervals were scored with the =STDEV.S and =CONFIDENCE functions on Microsoft Excel (Office 365).

Guidelines, expert opinion papers, commentaries, correspondences, studies exploring ethical concerns, reviews, systematic reviews and meta-analyses were examined.

### 2.1. Chromosomal Microarray Analysis

The search string was “(prenatal) AND ((chromosomal microarray) OR (array-CGH) OR (a-CGH) OR (CGH-array) OR (SNP-array))”. Papers which did not declare a proper ruling out of aneuploidies and gross chromosomal imbalances (>10 Mb) were secondarily excluded. The results were calculated as incremental yield over karyotyping. Diagnostics/VUSs rates were scored for the following:

- Group A: advanced maternal age, no indication of any chromosomal analysis, soft markers (single, multiple, pooled), NT (isolated), structural anomalies (single, multiple, pooled), dynamic anomalies, specific isolated soft markers, isolated structural anomalies subcategories, and isolated dynamic anomalies.
- Group B: soft markers (single, multiple, pooled), NT (pooled), structural anomalies (single, multiple, pooled), dynamic anomalies, specific isolated soft markers, isolated available structural anomalies subcategories, and isolated dynamic anomalies.

### 2.2. Exome Sequencing

We searched for (“WES” OR ‘Whole Exome Sequencing’ OR ‘Exome Sequencing’ OR “CES” OR “Clinical Exome Sequencing”) AND (‘Prenatal’). Papers not describing ES applications or describing ES performed after negative targeted panels were excluded. In all cases, karyotype and CMA were inconclusive. We collected the number of secondary and IFs in a unique category due to the ambiguous use of both terms in the literature.

Diagnostics/VUSs rates were scored for the following:

- Group A: the overall pooled anomalies and available subcategories.
- Group B: specific anomalies for which at least >2 papers were available. The analyzed categories included isolated and non-isolated cases of non-immune hydrops fetalis (NIHF), skeletal anomalies, congenital heart anomalies and isolated NT. When a single or only two papers were available, the diagnostic rates were annotated but not included in the meta-analysis.

### 2.3. Whole Genome Sequencing

We searched for ((WGS) OR ((whole genome sequencing)) OR (genome sequencing)) AND (prenatal). We excluded papers focusing on NIPS/NIPT. We divided the cohorts into two subgroups, distinguishing between whole genome sequencing (WGS) (≥20–30× depth of coverage) (Group 1) and low-coverage WGS (LC-WGS) (≤0.5× depth of coverage) (Group 2). Only prenatal cohorts with US anomalies were considered. When possible, we reclassified cases in two categories, multiple US anomalies and isolated US anomalies, recalculating diagnostic yield. In contrast, the overall diagnostic yield was considered. We reported or recalculated VUSs rate when data were available.

## 3. Results

### 3.1. Chromosomal Microarray

The literature research yielded 1370 results. A total of 253 papers were retained and analyzed, and 186 papers were deemed eligible for quantitative analysis. Among these, 71 described cohorts with pooled different indications (Group A), and 115 described cohorts enrolled for a specific single-district anomaly (Group B). In total, 7 articles were correspondences, 21 studies with ethics concerns, 4 guidelines, and 29 reviews.

Group A: A total of 40 were secondarily excluded from quantitative analysis [16,17,18,19,20,21,22,23,24,25,26,27,28,29,30,31,32,33,34,35,36,37,38,39,40,41,42,43,44,45,46,47,48,49,50,51,52,53,54,55]. Of these, 26 did not allow the scoring of the incremental diagnostic rate of CMA for the classes of fetuses selected for quantitative analysis [17,18,19,21,22,24,26,27,30,33,34,35,36,37,38,39,40,41,43,45,46,49,50,51,53,54,55], 6 did not allow proper exclusion of aneuploidies or gross chromosomal imbalances [23,32,42,47,48,52], 3 did not separate pathogenic results from VUSs [20,25,29], 3 used only targeted or low-resolution approaches [16,28,31], and 1 was a validation study on fetal samples with known diagnosis [44]. A total of 31 papers were eligible for quantitative analysis [11,56,57,58,59,60,61,62,63,64,65,66,67,68,69,70,71,72,73,74,75,76,77,78,79,80,81,82,83,84,85]. Of these, 20 were retrospective studies, 10 were prospective, and 1 did not declare pro- or retrospectivity. For six papers, the fetal sample was amniotic fluid. For one, the sample was fetal blood from cordocentesis. Twelve used amniotic fluid or chorionic villus, three used amniotic fluid or fetal blood, and nine used amniotic fluid, chorionic villus or fetal blood. Eleven of the selected papers explicitly reported IFs. It was not possible to perform statistical analysis on such data, as the definition of IFs varied from the identification of ROHs, from neurodevelopmental disorder susceptibility imbalances with reduced penetrance to pathogenic CNVs unrelated to the fetal phenotype. Diagnostic and VUSs rates for each paper are presented in Table S1. Results of the meta-analysis are shown in Table 1 and Table 2. The diagnostic rate for oligohydramnios, short femur, enlarged cisterna magna and mild pyelectasis and the VUS rate for hydrops, cystic hygroma, short femur and single umbilical artery were inferred from single sources. The average incremental diagnostic yield over karyotype for P/LP CNVs in advanced maternal age cases and in cases with no indication to chromosomal analysis was 0.84% (0.82–0.86) and 0.79% (0.74–0.83), respectively. The VUS rates were 1.75% (1.65–1.85) and 0.27% (0.24–0.30). These values should approximate the background risk for significant CNVs in low-risk pregnancies. There was a slight but significant increase in the diagnostic yield for soft markers. The yield was 2.15% (2.11–2.19) for isolated and 3.44% (3.24–3.64) for multiple soft markers. By pooling cohorts including both isolated and single soft markers, the yield was 2.45. (2.48–2.41).

For isolated NT, the rate of P/LP VUS was 2.63% (2.80–2.46), higher than isolated soft markers but lower than structural anomalies, and the VUS rate was 1.95% (1.82–2.08). When evaluating the diagnostic rate for structural anomalies, the yield was 3.66% (3.60–3.72) for single anomalies, 8.57% (7.92–9.22) for multiple anomalies and 5.72% (5.65–5.78) from cohorts including both single and multiple anomalies cases. VUSs yields for the same groups were 5.15% (5.05–5.26), 6.26% (5.95–6.57), and 2.86% (2.79–2.93). Concerning dynamic anomalies, the diagnostic and VUS rates were 4.90% (6.13–3.68) and not inferable for isolated fetal hydrops, 3.76% (4.31–3.20) and 25.00% (9.60–40.40) for cystic hygroma, 3.61% (3.88-3.34) and 4.98% (4.67–5.29) for amniotic fluid quantity anomalies, and 3.34% (3.52–3.17) and 6.35% (4.27–8.42) for FGR.

Group B: A total of 44 were secondarily excluded from quantitative analysis. Of these, 34 dealt with specific single-district malformations [86,87,88,89,90,91,92,93,94,95,96,97,98,99,100,101,102,103,104,105,106,107,108,109,110,111,112,113,114,115,116,117,118,119], 2 included postnatal cases [120,121], 3 dealt only with cases from fetuses’ demise or livebirths [122,123,124], 3 did not provide a proper distinction between US features [125,126,127], 1 dealt familial cases of heart disease [128], and 1 concerned twin pregnancies [129]. A total of 71 papers were eligible for quantitative analysis [6,130,131,132,133,134,135,136,137,138,139,140,141,142,143,144,145,146,147,148,149,150,151,152,153,154,155,156,157,158,159,160,161,162,163,164,165,166,167,168,169,170,171,172,173,174,175,176,177,178,179,180,181,182,183,184,185,186,187,188,189,190,191,192,193,194,195,196,197,198,199]: 44 were retrospective studies, 12 were prospective, and 15 did not declare pro- or retrospectivity.

Diagnostic and VUS rates for each paper are presented in Supplementary Table S2. The results of the meta-analysis are shown in Table 1 and Table 2. Single soft markers and single structural anomalies were two of the most consistently represented among all the studies, the former showing 5.30% (4.50–6.10) yield and the latter showing 5.64% (5.44–5.84) yield. The diagnostic rates for isolated mild ventriculomegaly, 6.95% (6.76–7.13), and for isolated short femur, 5.88% (5.37–6.40), were significantly higher than for other isolated soft markers. Concerning pooled NT, the rate of P/LP and VUS was 3.58% (3.47–3.68) and 2.96%. (2.88–3.04). In dynamic anomalies, the diagnostic and VUS rates were 3.28% (3.13–3.43) and 3.31% (3.09–3.54) for isolated FGR.

The most common incidental CNVs in both groups were *PMP22* (*6010979) deletions or duplications, leading to recurrent neuropathy with pressure palsies (MIM#162500) or Charcot–Marie–Tooth disease 1A (MIM#118220), respectively, and Xp22.31 deletions encompassing *STS* (MIM*300747), implied in X-linked ichthyosis (MIM#308100).

### 3.2. Exome Sequencing

The literature research yielded 989 results. Of these, 124 papers were retained, resulting in: 44 case studies, 36 reviews, 26 studies with ethics concerns, 4 seminars, 4 descriptive papers, 2 editorials, 2 comments, 3 correspondences, 2 position statements, and 1 committee opinion.

Group A: A total of 17 papers were included [13,75,200,201,202,203,204,205,206,207,208,209,210,211,212,213,214] (Table S3). Group B: A total of 27 cohorts were enrolled [98,196,215,216,217,218,219,220,221,222,223,224,225,226,227,228,229,230,231,232,233,234,235,236,237] (Table S4). For the quantitative analysis, 40 articles [121,238,239,240,241,242,243,244,245,246,247,248,249,250,251,252,253,254,255,256,257,258,259,260,261,262,263,264,265,266,267,268,269,270,271,272,273,274,275,276] were secondarily excluded. Of these, three papers focused on fetal demises or stillbirths [256,257,258], three papers focused on information postmortem [242,250,254], three were case reports [238,246,252], five focused on a single specific phenotype [240,241,248,261,274], three presented inhomogeneity in inclusion criteria and chromosomal anomalies/CNV assessment [239,251,262], six included both fetuses and postnatal cases [244,249,253,259,260,269], three focused on candidate genes [243,247,263], three focused on recurrent phenotypes or previously described cohorts [245,255,272], five were excluded for the lack of inclusion or eligibility criteria [264,265,268,271,276], two were excluded for the higher a priori risk for consanguinity and recurrence [266,270], one because parents were tested for recessive disorders [267], two because they focused on gene panels [273,275], and one due to the postnatal diagnosis [121].

All 17 papers from Group A and 17 from Group B were eligible for meta-analysis: three focusing on NIHF [215,216,217], six on isolated and non-isolated skeletal anomalies [218,219,220,221,222,223], two on isolated NT [196,225], and five on isolated and not isolated cardiovascular anomalies [231,232,233,234]. In one paper [224], isolated and non-isolated NT categories retrieved from other studies [201,202] were possible to separate, and the two categories were counted separately in the analysis.

Group A: The pooled incremental diagnostic yield over CMA for P/LP variants was 19.47% (18.94–20.01%), with 27.47% (26.45–28.49%) of diagnostic yield in the category of multiple structural anomalies (≥2) [13,75,200,201,202,203,204,205,206,207] (Table 3). The overall VUS rate was 8.32% (7.94–8.69%) (Table 3).

Data for the meta-analysis of the specific organ anomalies was not available for all the papers included in Group A. When data were available, most papers included in the anomaly-specific subcategories both fetuses with isolated features and associated anomalies, without the possibility to discern the two categories. For some of them with more than one anomaly, the count was repeated in several subcategories with variable inclusion criteria.

The pooled diagnostic rate was 14.77% (13.23–16.32%) in the group of any cardiovascular anomaly, 34.11% (31.42–36.80%) in musculoskeletal anomalies, 21.23% (17.49–24.97%) in genitourinary anomalies, 34.88% (30.21–39.55%) in craniofacial anomalies, 24.70% (23.94–25.45%) in CNS anomalies and 10.53% (7.53–13.52%) in abdomen or body wall anomalies. The subcategory of isolated NT when data were retrievable (6/17 papers) showed a diagnostic rate of 13.46% (10.19–16.74%). The diagnostic rate in the group of any dynamic anomalies was 20.78% (18.71–22.84%) (Table 3).

Group B: Most cohorts selected for specific anomalies presented both isolated and non-isolated anomalies (Table S4). When the articles were >2 for a specific category, we performed a meta-analysis to score diagnostic and VUS rates (Table 3). In the group of any NIHF (three papers, isolated and non-isolated), the pooled diagnostic yield was 30.81% (28.56–33.07%) and the VUS rate was 10.47% (9.14–11.79%) [215,216,217]. The diagnostic rate for skeletal dysplasia (six papers, isolated and non-isolated) was 70.19% (68.80–71.57%), whereas the VUS rate was 4.44% (3.67–5.22%) [218,219,220,221,222,223]. The rate for increased isolated NT (three papers—in one data, were retrieved from [224]) was 4.33% (3.59–5.07%), and the rate of VUS was 1.44% (0.46–2.42%) [196,224,225] (Table 3). The unique cohort of fetuses presenting non-isolated NT [224] showed a diagnostic rate of 26% and a VUS rate of 9% (Table S4). The rate for isolated or non-isolated cardiovascular anomalies (five papers) was 11.02% (10.65%–11.39%), whereas the VUS rate was 7.62% (7.48–7.76%) [231,232,233,234] (Table 3). In single papers describing specific anomalies, we reviewed articles annotating diagnostic yields. Concerning CNS, in a cohort of fetuses presenting isolated anomalies, the diagnostic rate was 45% [236]; while in another cohort of isolated and non-isolated CNS anomalies, the diagnostic rate was 50% [230]; and in a third cohort of fetuses presenting with isolated and not isolated cerebellar vermis defects and Dandy–Walker malformation, the diagnostic rate was 42% [98]. In two cohorts of fetuses presenting corpus callosum anomalies [227,228], the diagnostic rate scored 40% and 19%, whereas the VUS rate of 10% was reported in only one paper [228]. In a cohort of fetuses presenting isolated and non-isolated genitourinary anomalies, the diagnostic rate was 12% [235], whereas in another paper, the diagnostic rate was 7% [237]. In one recent paper analyzing fetuses with isolated hypoplastic/absent nasal bone, the diagnostic rate was 17% [277] (Table S4). Concerning IFs, 6 of the selected papers in Group A and 14 papers in Group B explicitly reported IFs (Tables S3 and S4). We did not score a specific rate due to the overly heterogeneous definition of IFs.

### 3.3. Whole Genome Sequencing

We obtained 353 results, with 41 meeting the initial inclusion criteria. Among these, 17 articles included prenatal cohorts, tested with different approaches after invasive sampling: 12 with LC-WGS [278,279,280,281,282,283,284,285,286,287,288,289] (Group 2) and 5 with WGS [290,291,292,293,294] (Group 1). In addition, 1 correspondence, 10 reviews, and 13 studies about ethical concerns were also considered. Within Group 2, seven articles were secondarily excluded due to inadequate inclusion criteria [279,281], mixed class of high-risk pregnancies and abnormal US findings [285], specific disease studied [284], highly specific analysis performed [282], validation nature of the study [280] and sole inclusion of postnatal cases [287]. Among Group 1, three articles were secondarily excluded due to the highly specific genomic analysis performed [291], highly specific structural anomaly studied [293], and not clearly reported results [294]. Two articles were retained for Group 1 [290,292] and five for Group 2 [278,283,286,288,289]. Overall, five studies were prospective, and two were retrospective. In one study [290], we revised the diagnostic yield, excluding four twin couples and a fetus that did not show US anomalies. We also divided the cases into isolated (single soft marker/structural anomaly/dynamic anomaly) and multiple (≥2 structural anomalies/dynamic anomalies) to compare the diagnostic yield of these two classes. In one study of Group 1 [292] and three studies of Group 2 [278,288,289], CMA or karyotype were applied contextually with WGS, comparing yields. In one work of Group 1 [290], a two-step strategy consisting of CMA plus ES, other than WGS as first-tier test, was applied. Conversely, in two studies of Group 2 [283,286], CMA and/or other tests were applied only to confirm variants detected with WGS. Due to the paucity of the studies, the different approaches applied, and the heterogeneity of their classifications, we forwent quantitative analysis for these approaches. Nevertheless, to compare results, we recalculated/reported yields in each study for the cohort of interest, and VUS when available (Table S5).

## 4. Discussion

### 4.1. Before Molecular Testing

Karyotyping is a staple for detecting chromosomal anomalies in both prenatal and postnatal diagnosis. It is based on the analysis of mitotic structures, displayed as pairs of homologous chromosomes. Karyotype allows detection of inversions, translocations, triploidies, chromosomal markers and numeric or structural anomalies in mosaicism state. The main limitations are the time required to culture cells and the resolution [295]. Rearrangements of 5 Mb can be identified if the Giemsa-banding pattern is sufficiently detailed in the region of interest at 400–550-band definition [14]. Chromosomal anomalies are detected in up to 8–10% of fetuses with US anomalies [296].

### 4.2. Chromosomal Microarray Analysis

CMA was introduced in prenatal diagnostics due to the incremental 10–15% yield over karyotype in children showing malformations, intellectual disability and dysmorphisms [297]. Despite CMA not being able to diagnose balanced chromosomal anomalies and low-level mosaicism, it is widely accepted as a first-tier test in fetuses with malformations [48,298]. It has gradually replaced karyotyping in several countries for other indications too [82,299,300,301]. The detection of submicroscopic CNVs allows the identification of a wider range of causes of malformations [302,303,304,305,306]. It represents a more standardized technique than karyotyping, being less prone to human errors. It has a markedly higher resolution and faster turnaround times, without requiring cell culturing [301]. Both aCGH and SNP-array platforms are comparable in the detection rate of significant CNVs [307]. It is a controversial question whether to universally perform SNP-arrays in prenatal settings to systematically detect partial or whole chromosome uniparental disomy or to identify ROH [27]. Up to 55% of fetuses with ROH present with US anomalies, frequently represented by malformations and FGR [308]. In these circumstances, ES might be encouraged to rule out autosomal recessive conditions. CNVs correlated with neurodevelopmental disorders can present highly variable clinical expressivity or incomplete penetrance [309]. It is important to point out during post-test counseling with the couple that these findings are usually inherited from an asymptomatic parent [310], but the parental phenotype does not predict the features of the child. It would be desirable for geneticists to be joined by psychologists, ensuring that the consultants are making the best decision for them [311]. Other potential findings can be implied in non-actionable late-onset diseases (i.e., neurological conditions, susceptibility to cancer development) [310] potentially relevant to parental health. Parental consanguinity or misattributed paternity (through ROHs detection) can be suspected too.

In the current meta-analysis, we found a background rate for P/LP submicroscopic CNVs pregnancies without indications for chromosomal analysis of 0.79%, comparable with the advanced maternal age cohort (0.84%). These rates appear to be lower than those initially reported, which ranged up to 1.7% [82]. The rate of diagnostic findings appeared to increase along with the severity of the conditions. Single soft markers presented with an overall diagnostic yield (2.15%) higher than baseline risk. However, each soft marker behaves differently, and the rate varies significantly between Group A and B. Diagnostic rates for soft markers were consistently higher in Group B, possibly due to selection bias of the latter. For example, the detection rate for intracardiac echogenic focus is 0.56% in Group A and 2.01% in B. Mild ventriculomegaly showed a diagnostic rate significantly higher than other soft markers in both groups, comparable to that of structural anomalies (Group A: 4.41%; B: 6.12%). Multiple soft markers also had the noticeably higher yield of 3.44%, overlapping that of single structural anomalies. Isolated short femur displayed even higher yields, 12.50% for Group A (single paper, 34 cases) and 5.88% in B (three papers, 187 cases). If these results are confirmed, it might be necessary to classify these anomalies as separated from traditional soft markers. Concerning NT, we could only score rates for isolated NT in Group A, but we could only pool NT cohorts including both associated and isolated cases for Group B, obtaining slightly different yields (Group A: 2.63%; B: 3.48%). In both cases, the yield was higher than soft markers and lower than structural anomalies. Dynamic anomalies were not pooled, being classified from exclusion from the other categories. Isolated FGR yielded similar diagnostic rates between the two groups (3.34%; 3.79%). Isolated polyhydramnios in Group A presented with a 3.18% diagnostic rate, while in Group B it accounts for 2.42%. The main difference between the two groups concerned structural anomalies. For example, cardiovascular malformations yielded 3.18% in Group A and 6.49% in B, even if both groups consisted of thousands of cases. The average detection rate of submicroscopic CNVs in cohorts enrolled for any single structural anomaly and normal karyotype from Group A was 3.66%. The diagnostic rate for multiple structural anomalies in the same group was 8.57%. For Group A, there was no marked difference between the diagnostic yields for different categories. In Group B, cardiovascular anomalies had the highest diagnostic rate, 6.47% when isolated, while genitourinary anomalies yielded the lowest, 3.35%. As expected, the diagnostic rates in each subcategory of Group B were much higher when associated malformations were present along with the index anomaly. The comparison of VUS rates does not allow proper conclusions, as the classification and report policies, as well as the availability of parental samples for segregation analysis, vary greatly. A high VUS rate in both groups was noted for FGR (Group A: 6.35%; B: 9.35%) and for CNS anomalies (7.55%; 8.56%). In other categories, VUS rates do not appear to differ greatly from the diagnostic rates. Some groups with high diagnostic rates, like multiple structural anomalies, also display high VUSs rates, in this specific case 6.25%. Given the results in the advanced maternal age and no indication of chromosome analysis groups, 1% might be an educated guess of the background VUS risk. This is consistent with reports from previous studies [312]. Low-resolution CMA reduces reported VUSs by 32% [313] but also fails to detect some P/LP CNVs [314]. The possibility of performing targeted or low-resolution microarray testing to protect consultants from VUSs misinterpretation, rather than a genome-wide one, has been extensively discussed [315]. Nowadays, genome-wide platforms are almost universally used when there are clinical indications to CMA [307]. Ideally, prospective parents should be allowed to decide whether to opt for targeted or genome-wide approaches, but it requires a cultural substrate that permits the consultants to understand advantages and disadvantages of these methods, making a conscious choice.

Indications to perform CMA in fetuses detected with soft markers are not standardized. In this meta-analysis, we highlighted that soft markers present very different rates of P/LP submicroscopic CNVs. Some often transient anomalies usually included among soft makers, such as short femur (Group A: 12.5%; B: 5.88%) and mild ventriculomegaly (4.41%; 6.95%), showed yield rates comparable to those of structural anomalies. These observations lead us to consider these findings as distinct entities from other soft markers.

### 4.3. Exome Sequencing

ES enables the simultaneous examination of a large number of genes in a limited turnaround time, with acceptable costs [316], presenting advantages especially in prenatal settings [317], where time represents a significant limit. It is based on massive parallel DNA sequencing, examining protein coding regions, which make up to 2% of the genome but include more than 85% of all variants responsible for monogenic conditions [318]. ES is able to detect single nucleotide variations, indels and, with specific software analysis not widely validated in diagnostic practice, CNVs [319].

In this meta-analysis in Group A, ES showed an overall diagnostic rate of 19.47% (18.94–20.01%), with 27.47% (26.45–28.49%) yield in multiple structural anomalies. We stratified the diagnostic yields by single-district anomalies retrieved from Group A and B. However, the classification of the anomalies and their associations had little consistency among studies, confounding the ability to obtain specific diagnostic rates per organ system. Studies with homogeneous inclusion criteria are needed to determine the specific diagnostic value of ES in single-district anomalies [279].

Deep phenotyping is essential for proper interpretation of variants and incomplete phenotype information in prenatal settings makes interpretation of variants challenging [279]. Predefined terms, such as human phenotype ontology (HPO), are useful to make the ES indications as univocal as possible [320], and a list of prenatal HPOs will be available shortly [321].

In this meta-analysis, the VUS rate was 8.32% in Group A, whereas it ranged between 1.44% and 10.47% in Group B. Although it is often left to the autonomy of individual laboratories, American College of Medical Genetics and Genomics (ACMG) guidelines recommend reporting VUSs related to autosomal recessive diseases when they are congruent with phenotype and a P/LP variant has been identified in the other allele [322].

In the current review, the IF rate was not assessable, due to the large heterogeneity in analyzing and reporting choices in the prenatal setting. ACMG detailed pretest considerations of reporting, IFs (both fetal and parental) and secondary findings, explicitly separating the terms [322]. Concerning postnatal IFs, ACMG suggests reporting only P/LP variants in 73 genes associated with “medically actionable” diseases [323]. The approach to these findings in prenatal diagnosis is still not defined.

### 4.4. Whole Genome Sequencing

WGS allows the sequencing of an individual’s whole genome. Potentially identifying more genetic variations than any targeted approach, WGS raises deep questions about interpretation and reporting of the variants. The reviewed cohorts (Table S5) applied two distinct strategies to the fetus. The first one is the high-coverage WGS, which explores anomalies ranging from chromosomal to single nucleotide variants (SNVs) in a single analysis. One study [290] applied a trio-based WGS to a heterogeneous population of fetuses affected by structural and dynamic anomalies. Multiple US anomalies yielded a higher diagnostic rate than isolated anomalies, which do not change significatively if we consider only associated structural anomalies (10/32, 31.3%), because of the small number of multiple dynamic anomalies in this class. In contrast, the diagnostic yield of isolated anomalies is 2.4% higher if we consider only structural findings (6/47, 12.8%). This is probably due to the high number of undiagnosed isolated dynamic anomalies (16/18, 94.4%). One study [292], applied singleton WGS to a cohort with increased NT, showing higher diagnostic yield in fetuses with NT plus associated anomalies. The overall diagnostic rate is higher compared to the previous WGS study, possibly due to the smaller sample size and to the lower heterogeneity of this second cohort. The remaining five studies applied low-coverage genome sequencing (LC-GS) [278,283,286,288,289]. Strong evidence supports that this approach, identifying gross and submicroscopic CNVs and chromosomal anomalies, could be applied as an alternative to CMA in prenatal diagnosis [278,279], requiring less DNA (thus reducing the need for cell culture) and performing better in regions with lower CMA probes density. Among these cohorts, three showed heterogeneous US anomalies [278,286,288], one showed soft markers [283], and one was enrolled for cardiac anomalies, isolated or not [289] (Table S5). The diagnostic yield of LC-GS is lower than WGS, as expected due to the different sensitivity. Internal comparison between WGS and the respective CMA results highlights a significantly higher diagnostic yield of the first approach [290,292], which is consistent with the WGS ability to also detect SNVs. In contrast, LC-GS displays less marked increase in the diagnostic yield than the gold standard approach [278], with a more noticeable increment if compared to karyotype [289] (Table S5). Finally, the overall VUSs number is consistently higher in the WGS cohort that reported this result [292] than in the LC-GS studies [283,289] (38% vs. 1.4% and 3.8%), potentially imputable to the higher number of variants detected with the first approach.

### 4.5. Diagnostic Yield Comparison: CMA and ES

A comparison between the diagnostic rates of CMA and ES is hindered by differences in enrollment criteria among cohorts from the two groups. The yield is scored as incremental over lower-tier testing, so all fetuses from ES cohorts had previously undergone CMA, with negative results. Further inhomogeneity arises from the different enrollment criteria specificities, varying from the type of anomaly to its possible association. For these reasons, a quantitative comparison can only be performed for a limited set of conditions (Table 4). Nevertheless, such analysis is of great interest, and some general broader assumptions can be inferred from the remaining data. The incremental diagnostic yield for fetuses enrolled for any structural anomaly, regardless of possible associations and involved organs, was 5.72% for CMA Group A, and 6.84% for CMA Group B, while ES Group A yielded 19.47%. The results inferred from the ES meta-analysis are consistently higher than those from CMA studies, and it is evident that the diagnostic yield of the ES is particularly promising in those cases in which the CMA has not been conclusive. For example, the ES diagnostic rate is 24.7% (Group A) for anomalies of the nervous system, compared to the 5.74% incremental yield of CMA over karyotype (Group B). As expected, both methods resulted in high diagnostic rates in fetuses with multiple structural anomalies, being 8.57 and 15.58 in CMA Group A and B, respectively, and reaching the value of 27.47% in ES Group A. No assumptions can be made on soft markers, as most of them are excluded from the ES cohort. Increased NT, which is sometimes considered a soft marker for chromosomal anomalies, is known to be associated with an increased risk of pathogenic CNV and of some monogenic disorders. The diagnostic yield in isolated increased NT was 2.63% in CMA Group A, and ranged from 13.6% in ES Group A to 4.33% in ES Group B. This suggests that an analysis for monogenic conditions can be advantageous in increased NT as well as in structural anomalies cases.

### 4.6. VUS Rate Comparison: CMA and ES

Further complications arise when comparing VUS rates, as the criteria for their classification and report varies greatly among studies. There are the same limitations described for the comparison between the diagnostic rates. A quantitative comparison is only available for pooled structural anomalies (2.86% and 3.34% in Group A and B via CMA versus 8.32% in Group B via ES), pooled cardiovascular anomalies (Group B: 5.56% via CMA versus 7.62% via ES) and isolated NT (1.95% in Group A via CMA versus 1.44% in Group B via ES) (Table 4). From these results, it is evident that in pooled structural anomalies, similarly to the greater diagnostic yield, ES presents a greater number of VUSs. As for cardiovascular anomalies and isolated NT, both techniques appear to reach comparable values.

### 4.7. Ethical Concerns

Each genetic test must be preceded and followed by non-directive counseling, aimed at educating consultants about the possible conditions related to the US picture and the array of available investigations, providing the basis for thoughtful decision making, according to psychological, socio-economic, cultural and religious backgrounds.

Interestingly, different communicative approaches have been reported depending on the type of society: where an individualistic culture prevails, both counselor and consultant are more assertive than more collectivistic countries [324]. Although genetic counseling must be non-directive, every geneticist is influenced by personal considerations on each single case, based on their experience or knowledge. It is essential that the geneticist shows empathy towards the couple without exposing himself excessively, not influencing the choices. Limited consultation time, different cultural backgrounds among patients and language barriers still represent great challenges [325]. The emotional state of the consultants, who often receive counseling immediately after the detection of the US anomaly, must also be considered. The resulting confusion and the disruption of the expectation of a healthy fetus can make it difficult to understand genetic implications and give true informed consent for testing. It is also essential to provide the basic communicative skills to the other health professionals, as fetal medicine is a multidisciplinary work, involving gynecologists, radiologists and pediatricians. Ideally, the communication should take place in a safe environment and in the presence of the whole team. Unfortunately, this clashes with practical concerns.

Reporting VUSs or IFs in prenatal settings is an extremely delicate question, and psychological support, often advocated but rarely proposed in actual clinical practice, should be considered [326]. VUSs are detected in about 5% of fetuses with structural anomalies via CMA and in around 8% of cases via ES (Table 2 and Table 3). These numbers inevitably increase when WGS is performed, due to the identification of VUSs also in the noncoding regions [290]. Consultants present different reactions after receiving information of unclear significance, which varies according to one’s own experiences, perceptions and family planning [326], and sometimes they feel reassured when inherited variants are found [327]. These findings can also negatively influence the doctor–patient relationship, if the potential VUSs and IFs identification has not been previously discussed [322,328].

These are among the most impacting findings in decision making, and prospective parents often do not have the time to evaluate what information they want to receive [329]. Indeed, there are no univocal protocols between the various countries, so there is great heterogeneity about what should be reported, who decides what to report [330], who must pay [331] and more.

Parental analysis is crucial for the interpretation of the results and for formulating recurrence risks. VUSs and IFs can present a clinical impact on both fetus and parents [332]. VUS carriers should be evaluated after birth [333] or if the couple is planning new pregnancies [334], even if no further phenotypic findings emerge. If the alteration is parentally inherited, instrumental analyses may be proposed for the carrier. This scenario opens up further ethical concerns. For example, it is sometimes impossible to undergo timely medical evaluations. The potential need for an out-of-pocket accession system for such assessments for a timely diagnosis introduces a different probability of reaching a correct interpretation of the variant based on the consultants’ economic status.

To complicate things, genetic findings are always reported under the mother’s name. Consequently, it can be impracticable to retrieve data during postnatal examinations [319,329]. As a final remark, negative results from genetic tests could be mistakenly interpreted by the consultants as necessarily reassuring findings [329], but it should be discussed that a negative analysis reduces the risk of a genetically determined condition without ever being able to exclude it.

### 4.8. Non-Invasive Prenatal Testing: From Screening to Diagnosis

After cell-free DNA (cfDNA) identification in maternal plasma, NIPT was developed to detect the most common fetal autosomal aneuploidies [335,336]. Later, sexual chromosomes aneuplodies analysis was developed, albeit with less reliable results. Today, NIPT is mainly performed with LC-GS (<0.5×), and recent development has focused on testing microdeletion/microduplication syndromes [337,338]. Nevertheless, more clinical validation studies are needed to test NIPT reliability on subchromosomal rearrangements before current use in clinical practice.

Some monogenic disorder assays have been considered for pregnancies at high risk for the specific condition as noninvasive prenatal diagnoses (NIPD), not requiring confirmation on fetal samples [339]. Paternally inherited or fetal de novo variants potentially causative for conditions with autosomal dominant inheritance are not present in the maternal genome. If the variant is detected in cfDNA, the fetus is predicted to be affected. Paternally inherited variants causing recessive disorders can be identified in the same way, if the partners carry different variants [340]. In this scenario, fetuses can present the paternal variant, being potentially affected or carrier, or not (unaffected). This approach can reduce the number of pregnant women undergoing invasive testing for known recessive conditions by a theoretical 50%. Relative mutation dosage is used when prospective parents carry the same recessive allele for maternally inherited dominant or X-linked pathogenic variants. It requires the exact quantification of the relative amount of reads. After linkage studies between the variant parental SNPs defining surrounding haplotypes, relative haplotype dosage [341,342] can be combined with relative mutation dosage, increasing the sensitivity.

### 4.9. RNA Sequencing

RNA-sequencing (RNA-seq) is an NGS-based technology that can sequence and quantitatively analyze RNA molecules in a biological sample. This approach can produce a plethora of valuable information on gene expression, novel transcript isoforms and allelic expression [343], which can help in characterizing cells, tissues and the human body. Due to the high amount of starting material needed and the restricted availability of fetal tissues, this approach has rarely been applied to prenatal fetuses, even for research purposes, causing an evident lack of data on prenatal tissues in the major gene expression atlases developed. Recently, the introduction of single-cell RNA-Seq, with a low amount of RNA required, has boosted the development of less invasive procedures which could in future be implemented in the molecular prenatal diagnosis and pregnancy monitoring, such as the detection of cell-free RNA in amniotic fluid [344], umbilical cord blood, placenta and maternal plasma [345].

### 4.10. Future Perspectives

The analysis of fetal DNA and RNA in maternal plasma, placenta and fetal fluids currently suffers from some issues, mainly due to the low quantity of fetal nucleic acids present, its mixed nature with maternal molecules, and technical and data analysis limitations. With further advances in WGS and RNA-seq techniques, the implementation of advanced sequencing platforms and bioinformatic analysis pipelines, as well as sequencing cost cutting, it can be foreseen that these less invasive approaches could be implemented in prenatal care and diagnosis, integrating additional valuable information on fetal health and complication of pregnancy [346]. In this view, the building of multidisciplinary teams of clinical and molecular geneticists and bioinformaticians could be essential to manage the interpretation of the enormous number of variants derived from the most advanced sequencing technologies in the time-efficient manner needed in prenatal diagnosis.

### 4.11. Diagnostic Workflow Suggestions

The definition of diagnostic workflows for prenatal diagnosis should not only consider diagnostic rate, but also turnaround time, VUSs management, economic factors and public health concerns. For fetuses with structural anomalies, CMA is universally considered a first-tier test, possibly after quantitative fluorescent polymerase chain reaction (for rapid detection of aneuploidies) negative results, to exclude aneuploidies [347]. This approach is undoubtedly valid, but we believe the information has to be integrated with structural chromosomal analysis and parental confirmations of the results. In this same class of fetuses, ES appears to have a very high incremental yield, at the cost of a significant VUSs burden. Given the economical, technical and interpretative limitations, a systematic application is not achievable yet. We encourage its use in cytogenetics-negative fetuses with multiple anomalies and in cases selected for familial history.

NT and fetal dynamic anomalies also benefit from the same approach as structural anomalies.

Regarding US minor findings, ES data are not available. We showed that soft markers present heterogeneous CMA results from each other. Short femur and mild ventriculomegaly present risks comparable to structural anomalies and should benefit from molecular analysis. For other isolated soft markers, the incremental yield is present but marginal, and a systematic recourse to CMA is far from unquestionable. The diagnostic rate of multiple soft markers poses a solid indication to CMA.

The moderate incremental yield over karyotyping of genome-wide CMA in low-risk pregnancies (advanced maternal age, parental anxiety) has unclear potential benefits due to the heterogeneity of possible results in fetuses without US anomalies, and a systematic application is not encouraged.

### 4.12. Limitations

Weaknesses of the literature search include the following:

- Non-homogeneous/standardized nomenclature of distinct molecular approaches in the literature, potentially limiting the efficiency of the search string in retrieving articles.

Weaknesses of the current meta-analysis include the following:

- Theoretically non-excludable heterogeneity in inclusion criteria of the cases chosen by the individual authors;
- Cases in which an associated malformation was not specified were considered to be affected by a single malformation;
- Diagnostic and VUS yields for specific soft markers were less reliable than US anomalies’ rates, due to smaller cohorts;
- Discrepancy of results explained by the different numerosity of the samples;
- Heterogeneity in molecular platforms.

## 5. Conclusions

Molecular approaches in the prenatal setting are critical tools for investigating the causes of fetal anomalies. From the current meta-analysis, it emerged that the overall diagnostic yield of mixed structural anomalies accounts for 5.72% for CMA and 19.47% for ES, with variability due to cohorts’ characteristics, the class of malformation and the numerosity of the samples. Among the most debated topics are the reporting and interpretation of VUSs in genes linked to the fetal phenotype and IFs. In this meta-analysis, VUSs reported for fetuses with structural anomalies were 2.86% for CMA and 8.32% for ES.

Interesting and original results emerged from the cohorts of fetuses with soft markers. We showed that soft markers present very different CMA diagnostic yields from each other, and some of them, including the short femur (Group A: 12.5%; B: 5.88%) and mild ventriculomegaly (Group A: 4.41%; B: 6.95%), have rates comparable to those of fetal structural anomalies. These observations lead us to consider some US findings as distinct entities from other soft markers. In our opinion, increased NT, short femur and mild ventriculomegaly should be considered like malformations, indicating the execution of CMA. Similarly, the detection of two or more soft markers should be managed in the same way as the detection of a structural anomaly. This information offers us new perspectives, and further studies are encouraged in order to definitively determine if all soft markers are truly “soft”.

## Figures and Tables

**Table 1:** CMA—Meta-analysis Group A and B. Diagnostic yield.

		Diagnostic Rate		Yield (%)		*n*° Papers	
Class	Status	Group A	Group B	Group A	Group B	Group A	Group B
Parental Anxiety/No Indication	-	78/9918	-	0.79 (0.74–0.83)	-	9	-
Advanced Maternal Age	-	135/16,083	-	0.84 (0.82–0.86)	-	10	-
**Structural Anomaly**	**Isolated**	**269/7352**	**297/5263**	**3.66 (3.60–3.72)**	**5.64 (5.44–5.84)**	**12**	**25**
**Structural Anomalies**	**Multiple**	**70/817**	**146/937**	**8.57 (7.92–9.22)**	**15.58 (14.57–16.59)**	**11**	**15**
**Structural Anomalies**	**Pooled**	**431/7540**	**107/1565**	**5.72 (5.65–5.78)**	**6.84 (6.44–7.23)**	**14**	**16**
Central Nervous System	Isolated	38/1067	14/327	3.56 (3.17–3.96)	4.28 (2.39–6.17)	7	2
Central Nervous System	Multiple	-	9/224	-	4.02 (3.57–4.47)	-	2
Central Nervous System	Pooled	-	36/627	-	5.74 (5.33–6.15)	-	4
Musculoskeletal	Isolated	42/891	-	4.71 (4.22–5.20)	-	8	-
Kidney/Genitourinary	Isolated	44/1269	39/1164	3.47 (3.21–3.72)	3.35 (3.26–3.44)	8	5
Kidney/Genitourinary	Pooled	-	24/421	-	5.70 (4.92–6.48)	-	5
Gastrointestinal	Isolated	4/86	-	4.65 (3.41–5.89)	-	5	-
Cardiovascular	Isolated	68/2139	229/3541	3.18 (3.11–3.25)	6.47 (6.23–6.71)	8	1
Cardiovascular	Multiple	-	113/586	-	19.28 (17.94–20.63)	-	1
Cardiovascular	Pooled	-	46/506	-	9.09 (8.22–9.97)	-	14
Thorax/Respiratory	Isolated	4/127	-	3.15 (2.72–3.58)	-	7	-
Craniofacial	Isolated	9/208	4/44	4.33 (3.79–4.86)	9.09	6	10
Craniofacial	Multiple	-	15/60	-	25.00	-	6
Abdomen/Body Wall	Isolated	3/90	-	3.33 (2.56–4.10)	-	2	-
Fetal Growth Restriction	Isolated	30/897	17/518	3.34 (3.17–3.52)	3.28 (3.13–3.43)	9	6
Fetal Growth Restriction	Multiple	-	18/201	-	8.96 (8.63–9.28)	-	3
Fetal Growth Restriction	Pooled	-	33/568	-	5.81 (5.59–6.02)	-	5
Amniotic Fluid Quantity	Isolated	21/582	-	3.61 (3.34–3.88)	-	6	-
Hydrops	Isolated	5/102	-	4.90 (3.68–6.13)	-	7	-
Polyhydramnios	Isolated	17/534	15/619	3.18 (2.95–3.42)	2.42	2	1
Polyhydramnios	Pooled	-	3/114	-	2.63	-	1
Oligohydramnios	Isolated	1/2	1/50	50.00 (50.00–50.00)	2.00	1	1
Cystic Hygroma	Isolated	13/346	-	3.76 (3.20–4.31)	-	3	-
**Soft Markers**	**Isolated**	**78/3633**	**47/887**	**2.15 (2.11–2.19)**	**5.30 (4.50–6.10)**	**6**	**10**
**Soft Markers**	**Multiple**	**22/639**	**6/132**	**3.44 (3.24–3.64)**	**4.55 (2.48–6.61)**	**4**	**2**
**Soft Markers**	**Pooled**	**107/4374**	**95/1407**	**2.45 (2.41–2.48)**	**6.75 (6.19–7.31)**	**6**	**8**
Echogenic Bowel	Isolated	2/216	5/242	0.93 (0.72–1.13)	2.07 (2.05–2.09)	4	2
Absent/Hypoplastic Nasal Bone	Isolated	2/99	16/165	2.02 (1.71–2.33)	9.70 (6.26–13.13)	3	3
Absent/Hypoplastic Nasal Bone	Pooled	-	27/122	-	22.13 (19.78–24.49)	-	3
Intracardiac echogenic Focus	Isolated	2/356	3/149	0.56 (0.33–0.79)	2.01 (1.77–2.25)	4	2
Intracardiac Echogenic Focus	Multiple	-	0/97	-	0.00	-	1
Intracardiac Echogenic Focus	Pooled	-	7/179	-	3.91	-	1
Choroid Plexus Cyst	Isolated	4/287	-	1.39 (1.30–1.49)	-	4	-
Choroid Plexus Cyst	Pooled	-	7/186	-	3.76	-	1
Enlarged Cisterna Magna	Isolated	0/10	-	0.00 (0.00–0.00)	-	1	-
Mild Pyelectasis	Isolated	0/25	-	0.00 (0.00–0.00)	-	1	-
Single Umbilical Artery	Isolated	0/37	-	0.00 (0.00–0.00)	-	2	-
Mild VentriculoMegaly	Isolated	9/204	23/331	4.41 (4.26–4.57)	6.95 (6.76–7.13)	3	3
Mild VentriculoMegaly	Pooled	-	64/968	-	6.61 (6.30–6.92)	-	4
Nuchal Translucency	Isolated	32/1217	-	2.63 (2.46–2.80)	-	9	-
Nuchal Translucency	Pooled	-	125/3495	-	3.58 (3.47–3.68)	-	14
Short Femur	Isolated	3/24	11/187	12.50 (12.50–12.50)	5.88 (5.37–6.40)	1	3
Short Femur	Multiple	-	9/67	-	13.43 (8.83–18.04)	-	2
Short Femur	Pooled	-	1/11	-	9.09	-	1

**Table 2:** CMA—Meta-analysis Group A and B. VUS rate.

		VUS Rate		Yield (%)		*n*° Papers	
Class	Status	Group A	Group B	Group A	Group B	Group A	Group B
Parental Anxiety/No Indication	-	9/3323	-	0.27 (0.24–0.30)	-	5	-
Advanced Maternal Age	-	217/12,388	-	1.75 (1.65–1.85)	-	6	-
**Structural Anomaly**	**Isolated**	**278/5394**	**144/3434**	**5.15 (5.05–5.26)**	**4.19 (3.86–4.53)**	**8**	**20**
**Structural Anomalies**	**Multiple**	**47/751**	**17/847**	**6.26 (5.95–6.57)**	**2.01 (1.67–2.34)**	**8**	**11**
**Structural Anomalies**	**Pooled**	**180/6288**	**39/1167**	**2.86 (2.79–2.93)**	**3.34 (3.03–3.66)**	**10**	**12**
Central Nervous System	Isolated	45/596	28/327	7.55 (6.40–8.70)	8.56 (5.53–11.59)	5	2
Central Nervous System	Multiple	-	2/224	-	0.89 (0.55–1.23)	-	2
Central Nervous System	Pooled	-	16/627	-	2.55 (2.27–2.83)	-	4
Musculoskeletal	Isolated	34/605	-	5.62 (5.17–6.07)	-	5	-
Kidney/Genitourinary	Isolated	63/1095	29/1164	5.75 (5.54–5.97)	2.49 (2.49–2.49)	5	5
Kidney/Genitourinary	Pooled	-	15/421	-	3.56 (3.56–3.57)	-	5
Gastrointestinal	Isolated	0/41	-	0.00	-	4	-
Cardiovascular	Isolated	40/1608	85/1830	2.49 (2.34–2.64)	4.64 (442–4.87)	5	10
Cardiovascular	Multiple	-	11/496	-	2.22 (1.97–2.47)	-	6
Cardiovascular	Pooled	-	6/108	-	5.56 (4.90–6.21)	-	2
Thorax/Respiratory	Isolated	0/18	-	0.00	-	4	-
Craniofacial	Isolated	0/23	0/44	0.00	0.00	3	1
Craniofacial	Multiple	-	0/60	-	0.00	-	1
Abdomen/Body Wall	Isolated	0/0	-	0.00	-	2	-
Fetal Growth Restriction	Isolated	37/583	16/483	6.35 (4.27–8.42)	3.31 (3.09–3.54)	5	5
Fetal Growth Restriction	Multiple	-	19/201	-	9.45 (8.13–10.78)	-	3
Fetal Growth Restriction	Pooled	-	26/479	-	5.43 (4.89–5.97)	-	3
Amniotic Fluid Quantity	Isolated	25/502	-	4.98 (4.67–5.29)	-	2	-
Hydrops	Isolated	0/0	-	0.00	-	1	-
Polyhydramnios	Isolated	25/494	-	4.25 (5.06–5.06)	-	2	-
Oligohydramnios	Isolated	0/0	2/50	0.00	4	-	1
Cystic Hygroma	Isolated	4/16		25.00 (9.60–40.40)	5.11 (4.68–5.53)	1	-
**Soft Markers**	**Isolated**	**58/3149**	**26/509**	**1.84 (1.81–1.87)**	**5.11 (4.68–5.53)**	**4**	**6**
**Soft Markers**	**Multiple**	**12/520**	**7/97**	**2.31 (2.18–2.44)**	**7.22**	**2**	**1**
**Soft Markers**	**Pooled**	**61/3412**	**16/593**	**1.79 (1.74–1.83)**	**2.70 (2.64–2.75)**	**4**	**4**
Echogenic Bowel	Isolated	2/133	9/242	1.50 (1.34–1.67)	3.72 (3.72–3.72)	3	2
Absent/Hypoplastic Nasal Bone	Isolated	3/99	5/118	3.03 (2.57–3.49)	4.24 (4.20–4.28)	2	2
Absent/Hypoplastic Nasal Bone	Pooled	-	2/52	-	3.85 (3.81–3.88)	-	1
Intracardiac echogenic Focus	Isolated	5/244	12/149	2.05 (1.76–2.34)	8.05 (7.09–9.02)	3	2
Intracardiac Echogenic Focus	Multiple	-	7/97	-	7.22	-	1
Intracardiac Echogenic Focus	Pooled	-	4/179	-	2.23	-	1
Choroid Plexus Cyst	Isolated	5/194	-	2.58 (2.16–3.00)	-	3	-
Choroid Plexus Cyst	Pooled	-	5/186	-	2.69	-	1
Enlarged Cisterna Magna	Isolated	0/0	-	0.00	-	-	-
Mild Pyelectasis	Isolated	0/0	-	0.00	-	-	-
Single Umbilical Artery	Isolated	1/37	-	2.70 (1.08–4.33)	-	1	-
Mild VentriculoMegaly	Isolated	8/264	-	3.03 (2.76–3.30)	-	2	-
Mild VentriculoMegaly	Pooled	-	5/189	-	2.65	-	1
Nuchal Translucency	Isolated	10/513	-	1.95 (1.82–2.08)	-	5	-
Nuchal Translucency	Pooled	-	72/2432	-	2.96 (2.88–3.04)	-	12
Short Femur	Isolated	1/24	2/187	4.17 (4.17–4.17)	1.07 (0.72–1.42)	1	3
Short Femur	Multiple	-	4/67	-	5.97 (3.49/8.45)	-	2
Short Femur	Pooled	-	2/11	-	18.18	-	1

**Table 3:** ES meta-analysis. Any refers to cohorts pooling both isolated and associated anomalies. NIHF: non-immune hydrops fetalis; CNS: central nervous system.

		Diagnostic Rate		Yield (%)		VUS Rate		Yield (%)		*n*° Papers	
Class	Status	Group A	Group B	Group A	Group B	Group A	Group B	Group A	Group B	Group A	Group B
**Pooled mixed anomalies**	**Any**	**384/1972**	**-**	**19.47 (18.94–20.01)**	**-**	**130/1563**	**-**	**8.32 (7.94–8.69)**	**-**	**17/17**	**-**
**Multiple Structural Anomalies**	**Associated (≥2)**	**211/768**	**-**	**27.47 (26.45–28.49)**	**-**	**-**	**-**	**-**	**-**	**17/17**	**-**
Cardiovascular anomalies ^†^	Any	39/264 ^†^	94/853	14.77 (13.23–16.32)	11.02 (10.65–11.39)	-	65/853	-	7.62 (7.48–7.76)	8/17	5
Musculoskeletal anomalies ^†^	Any	88/258 ^†^	-	34.11 (31.42–36.80)	-	-	-	-	-	9/17	-
Kidney/genitourinary anomalies ^†^	Any	31/146 ^†^	-	21.23 (17.49–24.97)	-	-	-	-	-	9/17	-
Skeletal dysplasias	Any	-	113/161	-	70.19 (68.80–71.57)	-	6/135	-	4.44 (3.67–5.22)	-	6
Craniofacial anomalies ^†^	Any	30/86 ^†^	-	34.88 (30.21–39.55)	-	-	-	-	-	6/17	-
CNS anomalies ^†^	Any	61/247 ^†^	-	24.70 (23.94–25.45)	-	-	-	-	-	10/17	-
Abdomen or body wall anomalies ^†^	Any	2/19 ^†^	-	10.53 (7.53–13.52)	-	-	-	-	-	4/17	-
**Dynamic anomalies ^†^**	**Any**	**32/154 ^†^**	**-**	**20.78 (18.71–22.84)**	**-**	**-**	**-**	**-**	**-**	**8/17**	**-**
**Nuchal Translucency**	**Isolated**	**14/104**	**9/208**	**13.46 (10.19–16.74)**	**4.33 (3.59–5.07)**	**-**	**3/208**	**-**	**1.44 (0.46–2.42)**	**6/17**	3 *
Non-Immune Hydrops Fetalis	Any	-	53/172	-	30.81 (28.56–33.07)	-	18/172	-	10.47 (9.14–11.79)	-	3

^†^ In the subcategories retrieved from the group of pooled mixed anomalies (cardiovascular, musculoskeletal, kidney/genitourinary, dynamic, craniofacial, CNS, abdomen or body wall anomalies), the count of fetuses with associated anomalies could be repeated in several subcategories. * in one retrieved from [224].

**Table 4:** CMA and ES comparison: detection rates and VUSs rates.

Anomaly	Status	Diagnostic Yield (%)				VUS Rate (%)			
		CMA		ES		CMA		ES	
		A	B	A	B	A	B	A	B
**Structural Anomaly**	Pooled	5.72 (5.65–5.78)	6.84 (6.44–7.23)	19.47 (18.94–20.01)		2.86 (2.79–2.93)	3.34 (3.03–3.66)	8.32 (7.94–8.69)	
Structural Anomaly	Multiple	8.57 (7.92–9.22)	15.58 (14.57–16.59)	27.47 (26.45–18.49)					
Central Nervous System	Pooled		5.74 (5.22–6.15)	24.7 (23.94–25.45)					
Cardiovascular	Pooled		9.09 (8.22–9-97)	14.77 (13.23–16.32)	11.02 (10.65–11.39		5.56 (4.90–6.21)		7.62 (7.48–7.76)
Genitourinary	Pooled		5.70 (4.92–6.48)	21.23 (17.49–24.97)					
**Nuchal Translucency**	Isolated	2.63 (2.46–2.80)		13.46 (10.19–16.74)	4.33 (3.59–5.07)	1.95 (1.82–2.08)			1.44 (0.46–2.42)

## Data Availability

The data presented in this study are available in the articles cited in the Reference Section.

## Supplementary Materials

The following supporting information can be downloaded at: https://www.mdpi.com/article/10.3390/diagnostics12030575/s1, Table S1: CMA—Group A Systematic review. P/LP = Pathogenic and Likely Pathogenic Variants. VUS = Variants of Uncertain Significance. NT = Nuchal Translucency. Hyb = hybrid CGH/SNP technology. The term “ANY” refers to cohorts including both isolated and associated cases. The rates are scored on cases without aneuploidies and gross (>10 Mb) Copy Number Variations. Table S2: CMA—Systematic Review—Group B. Table S3: ES systematic review Group A Any refers to cohorts pooling both isolated and associated anomalies. DR Diagnostic Rate. IFs (Incidental Findings) refer to both incidental and secondary findings. NT: Nuchal Translucency. CNS: Central Nervous System. Table S4: ES systematic review Group B Any refers to cohorts pooling both isolated and associated anomalies. IFs (Incidental Findings) refer to both incidental and secondary findings. NIHF (Non-Immune Hydrops Fetalis). NT: Nuchal Translucency. CNS: Central Nervous System. Table S5: WGS systematic review. DR: diagnostic rate. NT: nuchal translucency. IUGR: intrauterine growth restriction.
