# Supplementary material for: Molecular Approaches in Fetal Malformations, Dynamic Anomalies and Soft Markers: Diagnostic Rates and Challenges—Systematic Review of the Literature and Meta-Analysis
Source: Diagnostics (Basel). 2022 Feb 23;12(3):575. doi: 10.3390/diagnostics12030575 (PMC8947110; doi:10.3390/diagnostics12030575)
Supplement: Supplementary file 1 [file diagnostics-12-00575-s001.zip › diagnostics-1582110-SI.pdf]

Supplementary

**Table S1.** CMA - Group A Systematic review. P/LP= Pathogenic and Likely Pathogenic Variants. VUS= Variants of Uncertain Significance. NT= Nuchal Translucency. Hyb= hybrid CGH/SNP technology. The term “ANY” refers to cohorts including both isolated and associated cases. The rates are scored on cases without aneuploidies and gross (>10 Mb) Copy Number Variations.

| REF | test | No indication to karyotyping | | Advanced Maternal Age | | Single Soft Markers | | Multiple Soft Markers | | Any Soft Markers | | NT | | Single Structural Anomaly | | Multiple Structural Anomalies | | Any Structural Anomaly | | Dynamic Anomalies | |
|---|---|---|---|---|---|---|---|---|---|---|---|---|---|---|---|---|---|---|---|---|---|
| | | P/LP | VUS | P/LP | VUS | P/LP | VUS | P/LP | VUS | P/LP | VUS | P/LP | VUS | P/LP | VUS | P/LP | VUS | P/LP | VUS | P/LP | VUS |
| 56 | CGH | . | . | . | . | . | . | . | . | . | . | . | . | 2/44<br>4.45% | . | 1/8<br>12.50% | . | 4/69<br>5.80% | . | 1/34<br>2.94% | . |
| 58 | SNP | 3/321<br>0.93% | 0/321 | . | . | . | . | . | . | . | . | . | . | . | . | . | . | . | . | . | . |
| 57 | CGH | 4/391<br>1.02% | . | . | . | . | . | . | . | . | . | 4/93<br>4.30% | . | . | . | . | . | 5/84<br>5.95% | . | . | . |
| 59 | SNP | . | . | . | . | . | . | . | . | 6/359<br>1.67% | . | . | . | 16/215<br>7.44% | 4/215<br>1.86% | 5/22<br>22.72% | 2/22<br>9.09% | 21/237<br>8.86% | 9/237<br>3.80% | 6/140<br>4.28% | 2/120<br>1.67% |
| 60 | SNP | . | . | . | . | 13/401<br>3.24% | 14/401<br>3.49% | 13/382<br>3.40% | 11/382<br>2.88% | 26/710<br>3.66% | 25/710<br>3.52% | 11/284<br>3.87% | 7/284<br>2.46% | . | . | . | . | . | . | . | . |
| 61 | SNP | . | . | . | . | . | . | . | . | . | | . | . | 5/77<br>6.49% | 5/77<br>6.49% | 5/30<br>16.67% | 2/30<br>6.67% | 10/107<br>9.34% | 7/107<br>6.54% | . | . |
| 62 | CGH | 11/1647<br>0.67% | . | 6/1084<br>0.55% | . | . | . | . | . | . | . | . | . | . | . | . | . | . | . | . | . |
| 63 | CGH | . | . | . | . | . | . | . | . | . | . | . | . | 5/181<br>2.76% | 1/181<br>0.55% | 2/11<br>8.18% | 1/11<br>9.09% | 7/192<br>3.64% | 2/192<br>1.04% | . | . |
| 11 | SNP | . | . | . | . | 56/2252<br>2.49% | 34/2252<br>1.50% | 8/138<br>5.80% | 1/138<br>0.72% | 63/2390<br>2.63% | 35/2390<br>1.46% | . | . | . | . | . | . | . | . | . | . |
| 64 | SNP | . | . | 15/633<br>2.4% | 93/633<br>14.69% | . | . | . | . | . | . | . | . | . | . | . | . | . | . | 0/38 | . |
| 65 | CGH | . | . | . | . | . | . | . | . | . | . | 1/172<br>0.58% | . | 15/495<br>3.03% | . | . | . | . | . | 2/24<br>8.33% | . |
| 66 | hyb | . | . | 1/21<br>4.76% | . | . | . | . | . | . | . | . | . | . | . | . | . | . | . | . | . |
| 68 | SNP | 4/546<br>0.73% | . | . | . | 4/416<br>0.96% | . | 1/97<br>1.03% | . | 5/513<br>0.97% | . | . | . | . | . | . | . | . | . | . | . |
| 67 | CGH | 2/128<br>1.56% | 2/128<br>1.56% | . | . | . | . | . | . | . | . | . | . | . | . | . | . | . | . | . | . |
| 69 | SNP | . | . | . | . | . | . | . | . | . | . | . | . | 21/182<br>11.53% | 3/182<br>1.65% | 30/264<br>11.26% | 6/264<br>2.27% | 51/446<br>11.43% | 9/446<br>2.02% | . | . |
| 70 | SNP | . | . | 28/7588<br>0.37% | 63/7588<br>0.83 | 3/409<br>0.73% | 8/409<br>1.96% | . | . | . | . | 0/39 | 0/39 | 3/171<br>1.75% | 4/171<br>2.34% | 0/1 | 0/1 | 3/172<br>1.74% | 4/172<br>2.32% | 1/10<br>10.00% | 0/10 |
| 71 | SNP | . | . | . | . | 0/68 | . | 0/22 | . | 0/90 | . | . | . | 12/204<br>5.88% | . | 6/22<br>27.27% | . | 18/226<br>7.96% | . | 2/42<br>4.76% | . |
| 72 | SNP | 41/4174<br>0.98%% | . | | | . | . | . | . | . | . | . | . | . | . | . | . | . | . | . | . |
| 73 | both | . | . | 7/851<br>0.82% | 42/851<br>4.93% | . | . | . | . | . | . | . | . | . | . | . | . | . | . | . | . |
| 74 | CGH | 0/128 | 2/128<br>1.56% | 4/574<br>0.69% | 4/574<br>0.70% | . | . | . | . | . | . | 1/160<br>0.62% | 2/160<br>1.25% | . | . | . | . | 2/143<br>1.40% | 1/143<br>0.70% | . | . |
| 75 | SNP | . | . | . | . | . | . | . | . | . | . | . | . | 2/47<br>4.25% | 6/47<br>12.76% | 0/22 | 0/22 | 2/69<br>2.90% | 6/69<br>8.70% | 0/5 | 3/5<br>60.00% |
| 76 | CGH | 2/228<br>0.88% | . | . | . | . | . | . | . | . | . | . | . | . | . | . | . | . | . | 9/250<br>3.60% | . |

| | | | | | | | | | | | | | | | | | | | | | |
|---|---|---|---|---|---|---|---|---|---|---|---|---|---|---|---|---|---|---|---|---|---|
| 77 | CGH | . | . | . | . | . | . | . | . | . | . | 5/57<br>8.77% | . | . | . | . | . | . | . | . | . |
| 78 | CGH | . | . | . | . | . | . | . | . | . | . | . | . | 113/4319<br>2.61% | 243/4319<br>5.63% | 16/395<br>4.05% | 36/395<br>9.11% | 243/4319<br>5.62% | 129/4714<br>2.72% | 28/921<br>3.04% | 56/921<br>6.08% |
| 79 | both | 15/2746<br>0.54% | 5/2746<br>0.18% | 23/2318<br>0.99% | 15/2318<br>0.64% | . | . | . | . | 7/23<br>2.92% | 1/239<br>0.42% | . | . | . | . | . | . | . | . | . | . |
| 80 | both | . | . | . | . | . | . | . | . | . | . | 10/346<br>2.89% | . | 61/1133<br>5.38% | . | . | . | . | . | 12/282<br>4.25% | . |
| 81 | SNP | . | . | 13/624<br>2.08% | . | . | . | . | . | . | . | . | . | . | . | . | . | . | . | . | . |
| 82 | both | . | . | 34/1966<br>1.73% | . | . | . | . | . | . | . | . | . | . | . | . | . | 45/755<br>5.96% | . | . | . |
| 84 | CGH | . | . | . | . | | . | . | . | . | . | . | . | 4/82<br>4.88% | . | 5/36<br>13.89% | . | 9/118<br>7.62% | . | . | . |
| 83 | SNP | . | . | 4/424<br>0.94% | 0/424 | . | . | . | . | . | . | . | . | . | . | . | . | . | . | . | . |
| 85 | SNP | . | . | . | . | 2/87<br>2.30% | 2/87<br>2.30% | . | . | . | . | 0/30 | 1/30<br>3.33% | 10/202<br>4.95% | 13/202<br>6.43% | 0/6 | 0/6 | 10/208<br>4.80% | 13/208<br>6.25% | 2/59<br>3.4% | 1/36<br>2.78% |

**Table S2.** CMA – Systematic Review – Group B.

| REF | SINGLE SOFT MARKER | | MULTIPLE SOFT MARKERS | | ANY SOFT MARKER | | NT | | ISOLATED DYNAMIC ANOMALY | | DYNAMIC ANOMALIES ASSOCIATED | | ANY DYNAMIC ANOMALY | | SINGLE STRUCTURAL ANOMALY | | MULTIPLE STRUCTURAL ANOMALIES | | ANY STRUCTURAL ANOMALY | |
|---|---|---|---|---|---|---|---|---|---|---|---|---|---|---|---|---|---|---|---|---|
| | P/LP | VUS | P/LP | VUS | P/LP | VUS | P/LP | VUS | P/LP | VUS | P/LP | VUS | P/LP | VUS | P/LP | VUS | P/LP | VUS | P/LP | VUS |
| 6 | 0/47<br>0% | - | - | - | 3/35<br>8.57% | - | - | - | - | - | - | - | - | - | - | - | - | - | - | - |
| 130 | - | - | - | - | - | - | - | - | - | - | - | - | 4/70<br>5.71% | 4/70<br>5.71% | - | - | - | - | - | - |
| 131 | - | - | - | - | - | - | - | - | - | - | - | - | 7/127<br>5.51% | 2/127<br>1.57% | - | - | - | - | - | - |
| 132 | - | - | - | - | - | - | - | - | - | - | - | - | - | - | 5/149<br>3.36% | - | - | - | - | - |
| 133 | - | - | - | - | - | - | - | - | 4/84<br>4.76% | 0/84<br>0% | 5/49<br>10.2% | 0/49<br>0% | 9/133<br>6.77% | 0/133<br>0% | - | - | - | - | - | - |
| 134 | - | - | - | - | - | - | - | - | 0/78<br>0% | 3/78<br>3.85% | 4/65<br>6.15% | 3/65<br>4.62% | - | - | - | - | - | - | - | - |
| 135 | - | - | - | - | - | - | - | - | - | - | - | - | - | - | 4/101<br>3.96% | 1/101<br>0.99% | 11/26<br>42.31% | 2/26<br>7.69% | - | - |
| 136 | - | - | - | - | - | - | - | - | - | - | - | - | - | - | 7/103<br>6.80% | 3/103<br>2.91% | - | - | 4/17<br>23.53% | 1/17<br>5.88% |
| 137 | - | - | - | - | - | - | - | - | - | - | - | - | - | - | 4/125<br>3.20% | 4/125<br>3.20% | - | - | 2/22<br>9.09% | 3/22<br>13.64% |
| 138 | - | - | - | - | 7/186<br>3.76% | 5/186<br>2.69% | - | - | - | - | - | - | - | - | - | - | - | - | - | - |
| 139 | - | - | - | - | - | - | - | - | - | - | - | - | - | - | 8/305<br>2.62% | 18/305<br>5.90% | - | - | 10/194<br>5.15% | 5/194<br>2.58% |
| 140 | - | - | - | - | 7/176<br>3.98% | 5/176<br>2.84% | - | - | - | - | - | - | - | - | - | - | - | - | - | - |
| 141 | - | - | - | - | - | - | - | - | 1/35<br>2.86% | - | - | - | 1/89<br>1.12% | - | - | - | - | - | - | - |
| 142 | - | - | - | - | - | - | - | - | 3/62<br>4.84% | 4/62<br>6.45% | 9/87<br>10.34% | 16/87<br>18.39% | 12/149<br>8.05% | 20/149<br>13.42% | - | - | - | - | - | - |
| 143 | 6/98<br>6.12% | - | - | - | - | - | - | - | - | - | - | - | - | - | - | - | - | - | - | - |
| 144 | - | - | - | - | - | - | 11/599<br>1.84% | 8/599<br>1.34% | - | - | - | - | - | - | - | - | - | - | - | - |
| 145 | 3/139<br>2.16% | 6/139<br>4.32% | - | - | - | - | - | - | - | - | - | - | - | - | - | - | - | - | - | - |
| 146 | 3/86<br>3.49% | 1/86<br>1.16% | - | - | - | - | - | - | - | - | - | - | - | - | - | - | - | - | - | - |
| 147 | - | - | 6/35<br>17.14% | - | - | - | - | - | - | - | - | - | - | - | - | - | - | - | - | - |
| 148 | 3/141<br>2.13% | 12/141<br>8.51% | 0/97<br>0% | 7/97<br>7.22% | - | - | - | - | - | - | - | - | - | - | - | - | - | - | - | - |

| | | | | | | | | | | | | | | | | | | | | |
|---|---|---|---|---|---|---|---|---|---|---|---|---|---|---|---|---|---|---|---|---|
| **149** | 13/154<br>8.44% | - | - | - | - | - | - | - | - | - | - | - | - | - | - | - | - | - | - | - |
| **150** | - | - | - | - | - | - | - | - | - | - | - | - | - | - | 11/235<br>4.68% | 5/235<br>2.13% | 14/88<br>15.91% | 2/88<br>2.27% | - | - |
| **151** | 13/32<br>40.63% | 4/32<br>12.50% | - | - | 18/52<br>34.62% | 2/52<br>3.85% | - | - | - | - | - | - | - | - | - | - | - | - | - | - |
| **152** | - | - | - | - | 8/182<br>15.38% | - | - | - | - | - | - | - | - | - | - | - | - | - | - | - |
| **153** | 0/8<br>0% | 0/8<br>0% | - | - | 7/179<br>3.91% | 4/179<br>2.23% | - | - | - | - | - | - | - | - | - | - | - | - | - | - |
| **154** | - | - | - | - | - | - | - | - | - | - | - | - | - | - | 19/239<br>7.95% | 6/239<br>2.51% | - | - | - | - |
| **155** | - | - | - | - | - | - | - | - | - | - | - | - | - | - | - | - | 0/22<br>0% | 0/22<br>0% | - | - |
| **156** | - | - | - | - | - | - | 9/77<br>11.69% | 1/77<br>1.30% | - | - | - | - | - | - | - | - | - | - | - | - |
| **157** | - | - | - | - | - | - | 11/300<br>3.67% | 14/300<br>4.67% | - | - | - | - | - | - | - | - | - | - | - | - |
| **158** | - | - | - | - | - | - | - | - | - | - | - | - | - | - | 3/85<br>3.53% | 3/85<br>3.53% | - | - | 1/25<br>4% | 0/25<br>0% |
| **159** | - | - | - | - | - | - | - | - | - | - | - | - | - | - | 9/103<br>8.74% | 0/103<br>0% | 3/49<br>6.12% | 1/49<br>2.04% | - | - |
| **160** | - | - | - | - | - | - | - | - | - | - | - | - | - | - | 11/69<br>15.94% | - | 5/18<br>27.78% | - | 3/12<br>25% | - |
| **161** | - | - | - | - | - | - | - | - | - | - | - | - | - | - | 7/208<br>3.37% | 8/208<br>3.85% | - | - | 9/123<br>7.32% | 7/123<br>5.69% |
| **162** | - | - | - | - | - | - | - | - | - | - | - | - | - | - | 1/21<br>4.76% | 1/21<br>4.76% | 6/18<br>33.33% | 3/18<br>16.67% | 1/11<br>9.09% | 2/11<br>18.18% |
| **163** | - | - | - | - | - | - | 8/90<br>8.89% | 3/90<br>3.33% | - | - | - | - | - | - | - | - | - | - | - | - |
| **164** | - | - | - | - | - | - | - | - | - | - | - | - | - | - | - | - | 9/22<br>40.91% | - | 2/83<br>2.41% | - |
| **165** | - | - | - | - | - | - | - | - | - | - | - | - | - | - | - | - | 6/232<br>2.59% | 0/232<br>0% | - | - |
| **166** | - | - | - | - | - | - | 8/286<br>2.80% | 10/286<br>3.50% | - | - | - | - | - | - | - | - | - | - | - | - |
| **167** | - | - | - | - | - | - | - | - | 5/139<br>3.60% | 3/139<br>2.16% | - | - | - | - | - | - | - | - | - | - |
| **168** | - | - | - | - | - | - | - | - | - | - | - | - | - | - | 8/118<br>6.78% | - | - | - | 8/35<br>22.86% | - |
| **169** | - | - | - | - | - | - | 3/118<br>2.54% | 1/118<br>0.85% | - | - | - | - | - | - | - | - | - | - | - | - |
| **170** | - | - | - | - | - | - | - | - | 4/120<br>3.33% | 6/120<br>5% | - | - | - | - | - | - | - | - | - | - |
| **171** | - | - | - | - | - | - | 10/464<br>2.16% | - | - | - | - | - | - | - | - | - | - | - | - | - |

| | | | | | | | | | | | | | | | | | | | | |
|---|---|---|---|---|---|---|---|---|---|---|---|---|---|---|---|---|---|---|---|---|
| **172** | - | - | - | - | - | - | - | - | - | - | - | - | - | - | 31/331<br>9.37% | 15/331<br>4.53% | - | - | - | - |
| **173** | - | - | - | - | - | - | - | - | 15/619<br>2.42% | - | - | - | 3/114<br>2.63% | - | - | - | - | - | - | - |
| **174** | - | - | - | - | - | - | 9/599<br>1.50% | - | - | - | - | - | - | - | - | - | - | - | - | - |
| **175** | - | - | - | - | - | - | - | - | - | - | - | - | - | - | 18/1407<br>1.28% | - | - | - | 12/268<br>4.48% | - |
| **176** | - | - | - | - | - | - | - | - | - | - | - | - | - | - | - | - | 2/11<br>18.18% | - | - | - |
| **177** | - | - | - | - | - | - | 3/104<br>2.88% | 5/104<br>4.81% | - | - | - | - | - | - | - | - | - | - | - | - |
| **178** | 2/103<br>1.94% | 3/103<br>2.91% | - | - | - | - | - | - | - | - | - | - | - | - | - | - | - | - | - | - |
| **179** | - | - | - | - | - | - | - | - | - | - | - | - | 1/50<br>2% | 2/50<br>4% | - | - | - | - | - | - |
| **180** | - | - | - | - | - | - | - | - | - | - | - | - | - | - | 18/190<br>9.47% | 14/190<br>7.37% | - | - | - | - |
| **181** | - | - | - | - | - | - | - | - | - | - | - | - | - | - | 6/22<br>27.27% | 10/22<br>45.45% | 7/200<br>3.50% | 1/200<br>0.50% | 14/114<br>12.28% | 1/114<br>0.88% |
| **182** | - | - | - | - | - | - | 2/40<br>5% | 0/40<br>0% | - | - | - | - | - | - | - | - | - | - | - | - |
| **183** | - | - | - | - | - | - | 11/174<br>6.32% | 2/174<br>1.15% | - | - | - | - | - | - | - | - | - | - | - | - |
| **184** | - | - | - | - | - | - | - | - | - | - | - | - | - | - | 18/643<br>2.80% | 11/643<br>1.71% | - | - | 8/234<br>3.42% | 4/234<br>1.71% |
| **185** | - | - | - | - | - | - | - | - | - | - | - | - | - | - | - | - | 2/24<br>8.33% | 1/24<br>4.17% | 3/22<br>13.64% | 2/22<br>9.09% |
| **186** | - | - | - | - | - | - | - | - | - | - | - | - | - | - | 5/18<br>27.78% | 0/18<br>0% | - | - | 2/21<br>9.52% | 2/21<br>9.52% |
| **187** | 4/79<br>5.06% | - | - | - | - | - | - | - | - | - | - | - | - | - | - | - | - | - | - | - |
| **188** | - | - | - | - | - | - | - | - | - | - | - | - | - | - | 16/86<br>18.6% | - | 13/39<br>33.33% | - | - | - |
| **189** | - | - | - | - | - | - | - | - | - | - | - | - | - | - | 1/63<br>1.59% | 1/63<br>1.59% | - | - | - | - |
| **190** | - | - | - | - | 17/405<br>4.20% | - | - | - | - | - | - | - | - | - | - | - | - | - | - | - |
| **191** | - | - | - | - | - | - | 20/220<br>9.09% | 9/220<br>4.09% | - | - | - | - | - | - | - | - | - | - | - | - |
| **192** | - | - | - | - | - | - | - | - | - | - | - | - | - | - | 60/421<br>14.25% | 23/421<br>5.46% | 46/94<br>48.94% | 5/94<br>5.32% | 19/87<br>21.84% | 4/87<br>4.60% |
| **193** | - | - | - | - | - | - | - | - | - | - | - | - | - | - | 17/104<br>16.35% | 17/104<br>16.35% | - | - | - | - |
| **194** | - | - | - | - | - | - | - | - | - | - | - | - | - | - | - | - | - | - | 9/297<br>3.03% | 8/297<br>2.69% |

| | | | | | | | | | | | | | | | | | | | | | |
|---|---|---|---|---|---|---|---|---|---|---|---|---|---|---|---|---|---|---|---|---|---|
| **195** | - | - | - | - | - | - | - | - | - | - | - | - | - | - | 4/44<br>9.09% | 0/44<br>0% | 15/60<br>25% | 0/60<br>0% | - | - |
| **196** | - | - | - | - | - | - | 13/287<br>4.53% | 9/287<br>3.14% | - | - | - | - | - | - | - | - | - | - | - | - |
| **197** | - | - | - | - | 28/192<br>14.58% | - | - | - | - | - | - | - | - | - | - | - | - | - | - | - |
| **198** | - | - | - | - | - | - | 7/137<br>5.11% | 10/137<br>7.30% | - | - | - | - | - | - | - | - | - | - | - | - |
| **199** | - | - | - | - | - | - | - | - | - | - | - | - | - | - | 6/73<br>8.22% | 4/73<br>5.48% | 7/34<br>20.59% | 2/34<br>5.88% | - | - |

**Table S3. ES systematic review Group A** Any refers to cohorts pooling both isolated and associated anomalies. DR Diagnostic Rate. IFs (Incidental Findings) refer to both incidental and secondary findings. NT: Nuchal Translucency. CNS: Central Nervous System.

| REF. | Test | Overall DR (%) | DR (%) multiple structural anomalies | DR (%) any cardiovascular anomalies | DR (%) any genitourinary anomalies | DR (%) any musculo-skeletal anomalies | DR (%) any dynamic anomalies | DR (%) any craniofacial anomalies | DR (%) any CNS anomalies | DR (%) isolated NT | DR (%) any gastro-intestinal anomalies | DR (%) any respiratory/ thorax/chest anomalies | DR (%) any abdomen/ body wall anomalies | VUS (%) |
|---|---|---|---|---|---|---|---|---|---|---|---|---|---|---|
| **200** | WES | 37/94 (39.36%) | 13/34 (38.23%) | 1/12 (8.33%) | 4/11 (36.36%) | 15/23 (65.22%) | 0/1 | 3/6 (50.00%) | 1/6 (16.67%) | | . | . | 0/1 | 14/94 (14.89%) |
| **201** | WES in silico panel | 52/610 (8.52%) | 22/143 (15.38%) | 9/81 (11.11%) | 0/23 | 10/65 (15.38%) | 3/33 (9.09%) | . | . | . | . | . | . | 24/610 (39.34%) |
| **202** | WES | 24/234 (10.26%) | 14/74 (18.92%) | 4/77 (5.19%) | 4/25 (16.00%) | 8/34 (23.53%) | 3/29 (10.34%) | . | 11/49 (22.45%) | 6/51 (11.76%) | . | . | . | . |
| **203** | WES | 47/196 (23.98%) | 12/39 (30.77%) | 7/34 (20.59%) | 6/26 (23.08%) | 3/10 (30.00%) | . | 4/17 (23.53%) | 15/65 (23.08%) | . | 0/5 | . | . | 25/196 (12.76%) |
| **13** | CES | 46/133 (34.59%) | 39/91 (42.86%) | 11/37 (29.73%) | 12/38 (31.58%) | 28/72 (38.89%) | 23/64 (35.94%) | 22/48 (45.83%) | 22/64 (34.38%) | 4/11 (36.36%) | . | 9/21 (42.86%) | 2/15 (13.33%) | . |
| **204** | medical ES | 20/105 (19.04%) | 0/9 | 4/9 (44.44%) | 1/7 (14.29%) | 11/29 (37.93%) | 0/3 | 0/2 | 3/12 (25.00%) | 0/18 | . | . | 0/1 | 12/105 (11.43%) |
| **205** | WES | 16/85 (18.82%) | 7/18 (38.89%) | 2/10 (20.00%) | 1/8 (12.50%) | . | 0/7 | 1/9 (11.11%) | 2/11 (18.18%) | 3/8 (37.50%) | . | . | 0/2 | 16/85 (18.82%) |
| **206** | CES | 29/88 (32.95%) | 23/69 (33.33%) | . | . | 4/10 (40.00%) | . | . | 2/8 (25.00%) | . | . | . | . | 4/137 (2.91%) |
| **75** | CES | 27/80 (33.75%) | 8/22 (36.36%) | 1/4 (25.00%) | 3/4 (75.00%) | 9/13 (69.23%) | 2/13 (15.38%) | 0/4 | 2/11 (18.18%) | 1/4 (25%) | . | . | . | 5/80 (6.25%) |
| **207** | WES in silico panel | 6/14 (42.86%) | 6/14 (42.86%) | . | . | . | . | . | . | . | . | . | . | . |
| **208** | WES in silico panel | 7/15 (46.67%) | 7/15 (46.67%) | . | . | . | . | . | . | . | . | . | . | 2/15 (13.33%) |
| **209** | WES | 19/101 (18.81%) | 19/101 (18.81%) | . | . | . | . | . | . | . | . | . | . | 1/101 (0.99%) |

| | | | | | | | | | | | | | | |
|---|---|---|---|---|---|---|---|---|---|---|---|---|---|---|
| **210** | WES | 3/33 (9.09%) | 3/33 9.09%) | . | . | . | . | . | . | . | . | . | . | 6/33 (18.18%) |
| **211** | WES | 16/77 (20.78%) | 5/21 (23.81%) | . | . | . | . | . | 2/15 (13.33%) | 0/12 | . | . | . | . |
| **212** | WES | 8/20 (40.00%) | 8/20 (40.00%) | . | . | . | . | . | . | . | . | . | . | 1/20 (0.05%) |
| **213** | WES | 9/35 (25.71%) | 7/13 (53.85%) | . | 0/4 | 0/2 | 1/4 (25.00%) | . | 1/6 (16.67%) | . | . | . | . | 7/35 (20.00%) |
| **214** | CES | 18/52 (34.62%) | 18/52 (34.62%) | . | . | . | . | . | . | . | . | . | . | 13/52 (25.00%) |

**Table S4. ES systematic review Group B** Any refers to cohorts pooling both isolated and associated anomalies. IFs (Incidental Findings) refer to both incidental and secondary findings. NIHF (Non-Immune Hydrops Fetalis). NT: Nuchal Translucency. CNS: Central Nervous System.

| **Ref** | **Any NIHF** | | **Any skeletal anomalies** | | **Associated NT** | | **Isolated NT** | | **Any Diaphragmatic hernia** | | **Any CC anomalies** | | **Any Micrognathia** | | **Any CNS anomalies** | | **Isolated CNS anomalies** | | **Any Cardio-vascular anomalies** | | **Any genito-urinary anomalies** | | **Any cerebellar vermis defects and DWM** | | **Isolated Nasal Bone Absence or Hypoplasia** | |
|---|---|---|---|---|---|---|---|---|---|---|---|---|---|---|---|---|---|---|---|---|---|---|---|---|---|---|
| | **P/ LP** | **VUS** | **P/ LP** | **VUS** | **P/ LP** | **VUS** | **P/ LP** | **VUS** | **P/ LP** | **VUS** | **P/ LP** | **VUS** | **P/ LP** | **VUS** | **P/ LP** | **VUS** | **P/ LP** | **VUS** | **P/ LP** | **VUS** | **P/ LP** | **VU S** | **P/ LP** | **VUS** | **P/ LP** | **VUS** |
| **215** | 9/17 52.94 % | 4/17 23.53 % | . | . | . | . | . | . | . | . | . | . | . | . | . | . | . | . | . | . | . | . | . | . | . | . |
| **216** | 7/28 25.00 % | 2/28 7.14 % | . | . | . | . | . | . | . | . | . | . | . | . | . | . | . | . | . | . | . | . | . | . | . | . |
| **217** | 37/127 29.13 % | 12/127 9.45 % | . | . | . | . | . | . | . | . | . | . | . | . | . | . | . | . | . | . | . | . | . | . | . | . |
| **218** | . | . | 18/26 69.23 % | . | . | . | . | . | . | . | . | . | . | . | . | . | . | . | . | . | . | . | . | . | . | . |

| | | | | | | | | | | | | | | | | | | | | | | | | | | |
|---|---|---|---|---|---|---|---|---|---|---|---|---|---|---|---|---|---|---|---|---|---|---|---|---|---|---|
| **219** | . | . | 25/38<br>65.79 % | 4/38<br>10.53 % | . | . | . | . | . | . | . | . | . | . | . | . | . | . | . | . | . | . | . | . | . | . |
| **220** | . | . | 6/8<br>75.00 % | 0 | . | . | . | . | . | . | . | . | . | . | . | . | . | . | . | . | . | . | . | . | . | . |
| **221** | . | . | 35/55<br>63.63 % | 2/55<br>3.64 % | . | . | . | . | . | . | . | . | . | . | . | . | . | . | . | . | . | . | . | . | . | . |
| **222** | . | . | 23/26<br>88.46 % | 0 | . | . | . | . | . | . | . | . | . | . | . | . | . | . | . | . | . | . | . | . | . | . |
| **223** | . | . | 6/8<br>75 % | 0 | . | . | . | . | . | . | . | . | . | . | . | . | . | . | . | . | . | . | . | . | . | . |
| **224**<br>* | . | . | . | . | 24/91<br>26.37 % | 8/91<br>8.79 % | . | . | . | . | . | . | . | . | . | . | . | . | . | . | . | . | . | . | . | . |
| **224*** | . | . | . | . | . | . | 2/111<br>1.80 % | 0/111 | . | . | . | . | . | . | . | . | . | . | . | . | . | . | . | . | . | . |
| **196** | . | . | . | . | . | . | 3/24<br>12.50 % | 3/24<br>12.50 % | . | . | . | . | . | . | . | . | . | . | . | . | . | . | . | . | . | . |
| **225** | . | . | . | . | . | . | 4/73<br>5.48 % | 0 | . | . | . | . | . | . | . | . | . | . | . | . | . | . | . | . | . | . |
| **226** | . | . | . | . | . | . | . | . | 5/22<br>22.73 % | . | . | . | . | . | . | . | . | . | . | . | . | . | . | . | . | . |
| **227** | . | . | . | . | . | . | . | . | . | . | 2/5<br>40 % | . | . | . | . | . | . | . | . | . | . | . | . | . | . | . |
| **228** | . | . | . | . | . | . | . | . | . | . | 12/62<br>19.35 % | 6/62<br>9.68 % | . | . | . | . | . | . | . | . | . | . | . | . | . | . |
| **229** | . | . | . | . | . | . | . | . | . | . | | | 8/13 | . | . | . | . | . | . | . | . | . | . | . | . | . |

| | | | | | | | | | | | | | | | | | | | | | | | | | | |
|---|---|---|---|---|---|---|---|---|---|---|---|---|---|---|---|---|---|---|---|---|---|---|---|---|---|---|
| | | | | | | | | | | | | | 61.53 % | | | | | | | | | | | | | |
| **237** | . | . | . | . | . | . | . | . | . | . | . | . | . | . | . | . | 5/11<br>45.45 % | 0 | . | . | . | . | . | . | . | . |
| **230** | . | . | . | . | . | . | . | . | . | . | . | . | . | . | 3/6<br>50.00 % | 0 | . | . | . | . | . | . | . | . | . | . |
| **231** | . | . | . | . | . | . | . | . | . | . | . | . | . | . | . | . | . | . | 6/30<br>20.00 % | 2/30<br>6.67 % | . | . | . | . | . | . |
| **232** | . | . | . | . | . | . | . | . | . | . | . | . | . | . | . | . | . | . | 26/260<br>10.00 % | 16/260<br>6.15 % | . | . | . | . | . | . |
| **233** | . | . | . | . | . | . | . | . | . | . | . | . | . | . | . | . | . | . | 25/197<br>12.69 % | 10/197<br>5.07 % | . | . | . | . | . | . |
| **172** | . | . | . | . | . | . | . | . | . | . | . | . | . | . | . | . | . | . | 24/300<br>8.00 % | 32/300<br>10.66 % | . | . | . | . | . | . |
| **234** | . | . | . | . | . | . | . | . | . | . | . | . | . | . | . | . | . | . | 13/66<br>19.69 % | 5/66<br>7.58 % | . | . | . | . | . | . |
| **235** | . | . | . | . | . | . | . | . | . | . | . | . | . | . | . | . | . | . | | | 20/163<br>12.27 % | 2/163<br>1.23 % | . | . | . | . |
| **237** | . | . | . | . | . | . | . | . | . | . | . | . | . | . | . | . | . | . | . | . | 3/41<br>7.32 | 0 | . | . | . | . |

| | | | | | | | | | | | | | | | | | | | | | | | | | | |
|---|---|---|---|---|---|---|---|---|---|---|---|---|---|---|---|---|---|---|---|---|---|---|---|---|---|---|
| | | | | | | | | | | | | | | | | | | | | | % | | | | | |
| **98** | . | . | . | . | . | . | . | . | . | . | . | . | . | . | . | . | . | . | . | . | . | . | 8/19<br><br>42.11 % | 2/19<br><br>10.53 % | . | . |
| **239** | . | . | . | . | . | . | . | . | . | . | . | . | . | . | . | . | . | . | . | . | . | . | . | . | 2/12<br><br>16.67 % | |

**** splitted***

**Table S5. WGS systematic review**. DR: diagnostic rate. NT: nuchal translucency. IUGR: intrauterine growth restriction.

| *Ref.* | *Test* | *US anomaly/es* | *Overall DR* | *Overall VUS rate* | *Isolated anomaly DR* | *Isolated anomaly VUS rate* | *Multiple anomalies DR* | *Multiple anomalies VUS rate* | *Test performed other than WGS* | *Overall DR* | *Overall VUS rate* |
|---|---|---|---|---|---|---|---|---|---|---|---|
| | | | **P/LP** | | **P/LP** | | **P/LP** | | | **P/LP** | |
| **291** | trio based WGS (≈40x) | Structural or growth anomalies | 18/102 (17.6%) | NR | 7/67 (10.4%) | NR | 11/35 (31.4%) | NR | CMA + WES | CMA: 7/102 (6.9%) CMA + WES: 18/102 (17.6%) | NR |
| **293** | singleton WGS (≥ 30x) | NT isolated or not, ≥3.5 mm | 16/50 (32.0%) | 19/50 (38%) | 10/34 (29.4%) | 11/34 (32.4%) | 6/16 (37.5%) | 8/16 (50%) | CMA with/without kariotype | 8/50 (16.0%) | 6/50 (12%) |
| **279** | low coverage WGS | Structural abnormality or NT≥3.5 mm or IUGR | 55/503 (10.9%) | NC | -- | -- | -- | -- | CMA | 47/503 (9.3%) | NC |
| **284** | CNV-sequencing with low coverage WGS | US soft marker | 16/737 (2.2%) | 10/737 (1.4%) | -- | -- | -- | -- | Confirmatory studies (CMA, other) | -- | -- |
| **287** | trio based low coverage WGS | Abnormal US findings (structural and/or dynamic anomalies and/or increased NT) | 15/165 (9.1%) | -- | -- | -- | -- | -- | Confirmatory studies (CMA or qPCR) | -- | -- |
| **289** | CNV-sequencing with low coverage WGS | Isolated or multiple structural anomalies or isolated NT ≥ 3.5 mm or multiple US anomalies | 3/40 (7.5%) | -- | -- | -- | -- | -- | CMA | 3/40 (7.5%) | -- |
| **290** | low coverage WGS | Isolated or associated congenital heart disease | 28/181 (15.5%) | 7/181 (3.9%) | 14/146 (9.6%) | 7/146 (4.8%) | 14/35 (40%) | 0/35 (0%) | Karyotype | 20/181 (11.0%) | 0/181 (0%) |

NR: not reported; --: not evaluable; NC: not calculated
